# Pan-Cancer Study of SHC-Adaptor Protein 1 (SHC1) as a Diagnostic, Prognostic and Immunological Biomarker in Human Cancer

**DOI:** 10.3389/fgene.2022.817118

**Published:** 2022-05-02

**Authors:** Jianlin Chen, Gan Gao, Limin Li, Junping Ding, Xianhua Chen, Jianfei Lei, Haihua Long, Lihua Wu, Xin Long, Lian He, Yongqi Shen, Jinzhong Yang, Yonggang Lu, Yifan Sun

**Affiliations:** ^1^ Departments of Clinical Laboratory, Key Laboratory of medical molecular diagnostics of Liuzhou, Key Laboratory for nucleic acid molecular diagnosis and application of Guangxi health and wellness Commission, Affiliated Liutie Central Hospital of Guangxi Medical University, Liuzhou, China; ^2^ Departments of Clinical Laboratory of Liuzhou Maternity and Child Healthcare Hospital, Liuzhou, China; ^3^ Departments of Clinical Laboratory of Liuzhou People's Hospital, Liuzhou, China; ^4^ People’s Hospital of Rong’an County, Liuzhou, China

**Keywords:** SHC1, pan-cancer, tumor immunity, TCGA, biomarker, prognosis

## Abstract

**Background:** Recent studies highlight the carcinogenesis role of SHC-adaptor protein 1 (SHC1) in cancer initiation, development, and progression. However, its aberrant expression, diagnostic and prognostic value remain unknown in a variety of tumors.

**Methods:** The SHC1 expression profiles were analyzed using GTEx database, TCGA database, Oncomine and CPTAC database. The survival analysis was conducted using GEPIA2, Kaplan-Meier Plotter, UALCAN, and PrognoScan. The diagnostic values of SHC1 were calculated with the “pROC” package in R software. The genetic alteration of SHC1 and mutations were analyzed using cBioPortal. TIMER2 was employed to estimate the correlations between SHC1 expression and tumor-infiltrating immune cells in the TCGA cohort. Enrichment analysis of SHC1 was conducted using the R package “clusterProfiler.”

**Results:** SHC1 was ubiquitously highly expressed and closely associated with worse prognosis of multiple major cancer types (all *p* < 0.05). Further, SHC1 gene mutations were strongly linked to poor OS and DFS in SKCM (all *p* < 0.05). An enhanced phosphorylation level of SHC1 at the S139 site was observed in clear cell RCC. Additionally, the results revealed SHC1 expression was strongly linked to TMB, MMRs, MSI, TAMs, DNA methylation, m6A RNA methylation, tumor-associated immune infiltration, and immune checkpoints in multiple cancers (all *p* < 0.05). In addition, the results of the ROC analysis indicated the SHC1 exhibited strong diagnostic capability for KICH (AUC = 0.92), LIHC (AUC = 0.95), and PAAD (AUC = 0.95). Finally, enrichment analysis indicated that SHC1 may potentially involve in the regulation of numerous signaling pathways in cancer metabolism and protein phosphorylation-related functions.

**Conclusions:** These findings highlight that SHC1 plays an important role in the tumor immune microenvironment, and SHC1 has been identified to have prognostic and diagnostic value in multiple cancers. Thus, SHC1 is a potential target for cancer immunotherapy and effective prognostic and diagnostic biomarker.

## Introduction

Tumorigenesis is a complex process that makes cancer treatment difficult. Pan-cancer analysis has gained popularity due to its comprehensive and systematic research pattern, and the analysis can be conducted using databases such as The Cancer Genome Atlas (TCGA) (Blum et al., 2018) and Gene Expression Omnibus (GEO) (Clough, 2016), which contain functional genomics data sets from various malignancies.

SHC1, a gene implicated in tumorigenesis, encodes three major isoforms (p52ShcA, p46ShcA, and p66ShcA) with distinct functions and subcellular locations (Pelicci et al., 1992a). Two isoforms, p52ShcA and p46ShcA, are involved in signal transduction of receptor tyrosine kinase, and p66SHCA is engaged in cell senescence, apoptosis, and oxidative stress (Falco et al., 2005; Choi et al., 2015; Ahn et al., 2017a). Previous research demonstrated that high SHC1 expression predicts poor survival in hepatocellular carcinoma (HCC) patients (Ahn et al., 2017a). In breast cancer, the p52ShcA isoform acts as a promoter and plays a key role in 7,12-dimethylbenz(a)anthracene-induced tumorigenesis (Hudson et al., 2014a). Additionally, a recent study found that targeting SHC1 with mir-5582-5p induced cell apoptosis and cycle arrest in colorectal cancer (Cho et al., 2016). In a different study it was revealed that RAB14 has a carcinogenic effect in bladder cancer by downregulating SHC1 expression (Chao et al., 2019a). Moreover, Lai et al. (Lai et al., 2020a) demonstrated that SHC1 targeting DEPDC1B promotes the progression of bladder cancer. However, to date, there are no large-scale pan-cancer analyses and systematic studies on the relationship between SHC1 expression and significant clinical outcomes in various tumor types.

Human SHC1 (SHC-Adaptor Protein 1), family of adapter proteins, consists of three isoforms which integrate and transduce external stimuli to different signaling networks (Mir et al., 2020). SHC1 plays a key role in proliferation and tumorigenesis through transcriptional activation of downstream signal cascades such as RAS/MAPK and PI3K (Campbell et al., 1994; Lai et al., 2020b). Increased SHC1 gene expression and its transcriptional signature are detected in many cancer types (Miller et al., 2019; Lewis et al., 2020; Zhao et al., 2020; Liang et al., 2021). Notably, bioinformatics analysis revealed that high expression of SHC1 as a prognostic factor displayed worse prognosis in many cancer types (He et al., 2019; Huang et al., 2019; Morais-Rodrigues et al., 2020; Liang et al., 2021). Recent studies also found that SHC1 involved in tumor microenvironment and was a promising immunotherapy target for cancer treatment (Huang et al., 2019; Carrato et al., 2020; Hu et al., 2020; Zhao et al., 2020; Yang et al., 2021). Therefore, it is critical to implement a pan-cancer analysis to understand SHC1 copy number, mRNA and protein expression, and to evaluate its association with clinical outcomes and latent molecular mechanisms in cancer therapy.

In this study, we comprehensively investigated, for the first time, the SHC1 expression characteristics using a pan-cancer analysis of multi-database. Moreover, we investigated the relationship between SHC1 expression and TMB, MMRs, MSI, DNA methylation, m6A RNA methylation, protein phosphorylation, tumor immune infiltration, common immune checkpoint, and tumor-associated macrophage (TAM) from the related public websites. Finally, we evaluated the potential diagnostic value of SHC1 across cancers to understand its underlying mechanisms in pathogenesis and clinical course of cancer.

## Materials and Methods

### SHC1 Expression Analysis in Human Pan-Cancer

The Oncomine database (Rhodes et al., 2007) was used to investigate the variations in SHC1 expression between various types of cancer tissues and normal tissues. Then, TIMER2.0 and GEPIA2.0 were employed to verify SHC1 mRNA levels in 33 cancers based on TCGA expression profile data and the GTEx database (Tang et al., 2019). Additionally, the expression of SHC1 in different pathologic stages of all tumors was analyzed using the “Pathological Stage Plot” module of GEPIA2. All expression data were normalized using log2 [TPM (Transcripts per million) +1] transformation.

The UALCAN portal of CPTAC dataset was used to explore the levels of total protein or phosphoprotein (with phosphorylation at the Y428, S454, and S139 sites) of SHC1 (NP_001123512.1) between the normal tissues and the corresponding datasets of six tumor tissues (Chandrashekar et al., 2017).

### Survival and Prognosis Analysis

The correlation between SHC1 gene expression and the survival data in pan-cancer patients was evaluated using GEPIA2.0, Kaplan-Meier Plotter, UALCAN, and PrognoScan (Mizuno et al., 2009; Lánczky et al., 2016; Chandrashekar et al., 2017; Tang et al., 2019). We first obtained the significance map of SHC1 in 33 cancer types, including overall survival (OS) and relapse-free survival (RFS), using the “Survival Map” module of GEPIA2. Then, the relationship between SHC1 expression and OS or RFS in 21 cancer types was evaluated using the “Pan-cancer RNA-seq” module of Kaplan-Meier Plotter. The medians of SHC1 expression values were used as thresholds for bifurcating the low and high-expression groups. Finally, we used the UALCAN web tool and PrognoScan to evaluate the correlation between SHC1 expression and survival outcome in various cancers. In all analyses, statistical significance was determined using *p* < 0.05.

### cBioPortal Data, TMB, and MSI Analyses

The status of gene mutation in SHC1 was investigated using cBioPortal web tool (Gao et al., 2013). The “Cancer Types Summary” module was used to collect mutation characteristics such as changes in copy number, mutation rates or type in pan-cancers. The mutated sites were further examined using the “Mutations” module of cBioPortal. Furthermore, we used the “Comparison” module to compare the data on OS, DSS, and progress free survival (PFS) across all TCGA tumors with or without SHC1 gene mutations. The correlation between SHC1 gene expression and TMB or MSI across different tumors of TCGA was assessed using Spearman’s test and represented *via* the R “fmsb” package.

### Correlation Between SHC1 Expression and Various Gene Modifications

The TIMER2.0 and GEPIA2.0 databases were employed to determine the correlation between SHC1 expression, DNA mismatch repair system (MMRs) genes, DNA methyltransferase genes, and m6A RNA modification regulators in pan-cancer. The MMRs are composed of five genes: MLH1, MSH2, MSH6, PMS2, and EPCAM; the DNA methyltransferase genes include DNMT, DNMT3A, and DNMT3B; and the m6A RNA modification regulator is composed of 30 genes including ABCF, ALKBH5, CBLL1, EIF3A, EIF4G2, ELAVL1, FTO, FXR1, FXR2, G3BP1 and others. All the correlation analyses were performed using the Spearman’s correlation method.

### Immune Characteristics Analysis

The relationship between SHC1 expression and various types of tumor-infiltrating immune cells, such as B cells, CD4 (+) T cells, CD8 (+) T cells, macrophages, dendritic cells, neutrophils, and tumor-associated macrophages (TAMs) was explored across all TCGA tumors using the “Immune-Gene” module of TIMER2.0 (Aran and Butte, 2016). Both the TIMER and CIBERSORT algorithms were used to estimate the immune infiltration levels of various types of tumor-infiltrating immune cells. Next, we used the “Gene_Outcome” module of TIMER2.0 to evaluate the significance of gene expression in clinical outcome. The Spearman’s correlation method was used to evaluate the correlations between SHC1 expression, the immunological checkpoint markers, TAMs markers, M1 and M2 macrophages through TIMER2 and GEPIA2.

### Diagnostic Analysis

SHC1 expression data was downloaded from the GTEx and TCGA databases. The RNA-seq data in TPM format were analyzed after log2 conversion. The diagnostic values of SHC1 were calculated using “pROC” packages (Robin et al., 2011) in R version 3.6.3. The ROC curves were visualized using the “ggplot2” package (Wickham, 2016).

### Pathway and Enrichment Analysis

The top 50 SHC1-correlated binding proteins were first selected using the STRING to gain the protein network interaction diagram (Cui et al., 2020a). In addition, the “correlation analysis” module was also used to obtain the top 100 SHC1-associated genes according to *p* values from the GEPIA2. The six most relevant genes were selected from the above-mentioned 150 genes, based on their correlation coefficient. Afterwards, we used GEPIA2 to assess gene correlation between SHC1 and selected genes and create a correlation scatter diagram. Moreover, we used TIMER2 web-based tool to generate a heat map of the relationship between the six selected genes and SHC1 in 32 cancers. Furthermore, the Venn diagram viewer (Bardou et al., 2014) was used to perform an intersection analysis of the SHC1-binding and interacted genes. Finally, the two sets of 150 total genes from GEPIA2 and TIMER2 were imported into enrichment analysis. The statistical analysis and visualization of KEGG and GO analysis were all performed in “clusterProfiler” and the “GGplot2” packages in R (version 3.6.3) (Yu et al., 2012).

### Statistical Analysis

All correlation analyses in this study were performed by the Spearman correlation analysis. All statistical analyses were performed through R 3.6.3 Software. *p* < 0.05 was regarded as statistically significant.

## Results

### The Aberrant Expression Levels of SHC1 in Pan-Cancer

**FIGURE 1:**
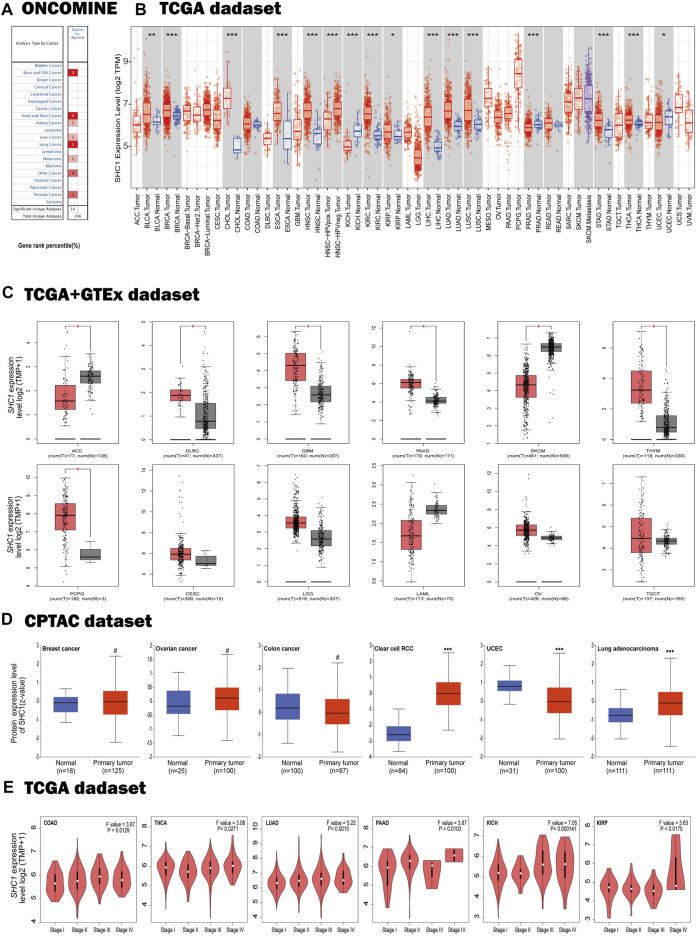
Expression profiles of SHC1 in tumors at different pathological stages. **(A)** Increased SHC1 expression of different cancers versus normal tissues from the Oncomine database. **(B)** The mRNA expression profiles of SHC1 in different cancers determined using TIMER2.0 (*p < 0.05; **p < 0.01; ***p < 0.001). **(C)** ACC, DLBC, GBM, PAAD, SKCM, THYM, CESC, LGG, LAML, OV, SARC, and TGCT in the TCGA database and the controls from the GTEx database. Data were expressed as box plots. (*p < 0.01) **(D)** Boxplot results of the expression levels of SHC1 in breast cancer, ovarian cancer, colon cancer, clear cell RCC and UCEC analyzed using the CPTAC dataset. (^#^ *p* > 0.05, ***p < 0.001). **(E)** The mRNA values of SHC1 of COAD, THCA, LUAD, PAAD, KICH, and KIRP evaluated at the main pathological stages (stage I, stage II, stage III, and stage IV) using the GEPIA2. Log2 (TPM+1) was used for log-scale.

To evaluate differences in SHC1 expression in various tumor and normal tissues, the levels of SHC1 mRNA were investigated using the Oncomine database. The expression of SHC1 was found to be significantly upregulated in brain and CNS, head and neck, kidney, liver, lung, skin, and prostate tumor samples compared to the normal samples (Figure 1A, *p*-value = 0.05, fold change = 2). The results of SHC1 expression are described in detail in Table 1. In addition, the SHC1 gene expression across various cancer types in the TCGA were evaluated using TIMER2. The mRNA expression level of SHC1 was higher in tumor tissues of BLCA (*p* < 0.01), BRCA, CHOL, ESCA, HNSC, KICH, LIHC, LUAD, LUSC, STAD, THCA (*p* < 0.001), and KIRP (*p* < 0.05) than in the normal tissues. However, SHC1 expression was found to be significantly lower in KIRC, PRAD (*p* < 0.001), and UCEC (*p* < 0.05) than in the corresponding normal tissues.

**TABLE 1:** SHC1 expression in cancers verus normal tissue in Oncomine database.

Cancer	Cancer type	*p*-value	Fold change	Rank (%)	Sample	Reference (PMID)
Brain and CNS	Glioblastoma	1.20E−19	2.815	1	180	16616334
	Glioblastoma	4.57E−7	2.464	3	42	12894235
	Glioblastoma	6.52E−6	2.943	6	54	16204036
Head and neck	Tongue Squamous Cell Carcinoma	1.05E−12	3.020	1	58	19138406
	Tongue Squamous Cell Carcinoma	5.24E−12	2.560	2	93	15833835
Kidney	Clear Cell Renal Cell Carcinoma	2.60E−5	3.514	6	20	17699851
Liver	Hepatocellular Carcinoma	1.31E−5	2.172	9	43	21159642
Lung	Squamous Cell Lung Carcinoma	2.86E−12	2.537	1	93	15833835
Skin	Cutaneous Melanoma	6.78E−5	4.625	10	70	16243793
Prostate	Prostate Carcinoma Epithelia	9.14E−5	2.059	5	101	17173048
Other	Mixed Germ Cell Tumor	2.18E−12	2.361	2	107	16424014
	Embryonal Carcinoma	6.23E−9	2.200	3	107	16424014
	Yolk Sac Tumor	2.56E−5	3.031	6	107	16424014
	Teratoma	2.78E−6	2.286	9	107	16424014

Using the GTEx dataset as controls and the TCGA data as tumor groups, we discovered an aberrant difference in SHC1 expression between tumor tissues and normal tissues in ACC, DLBC, GBM, PAAD, PCPG, SKCM, and THYM (Figure 1C, *p* < 0.01). However, no significant differences were recorded in CESC, LGG, LAML, OV, SARC, or TGCT. The results from the CPTAC dataset further confirmed that total protein of SHC1 was significantly higher clear cell RCC, LUAD (Figure 1D, *p* < 0.001), and lower in UCEC (*p* < 0.001) than in the corresponding normal tissues. Furthermore, the SHC1 mRNA levels correlated to pathological stage of COAD, THCA, LUAD, PAAD, KICH, and KIRP cancer (Figure 1E, *p* < 0.05). Taken together, these results strongly suggested that the SHC1 gene was abnormally regulated in multiple cancers as opposed to normal tissues.

### Multifaceted Prognostic Value of SHC1 in Cancers

**FIGURE 2:**
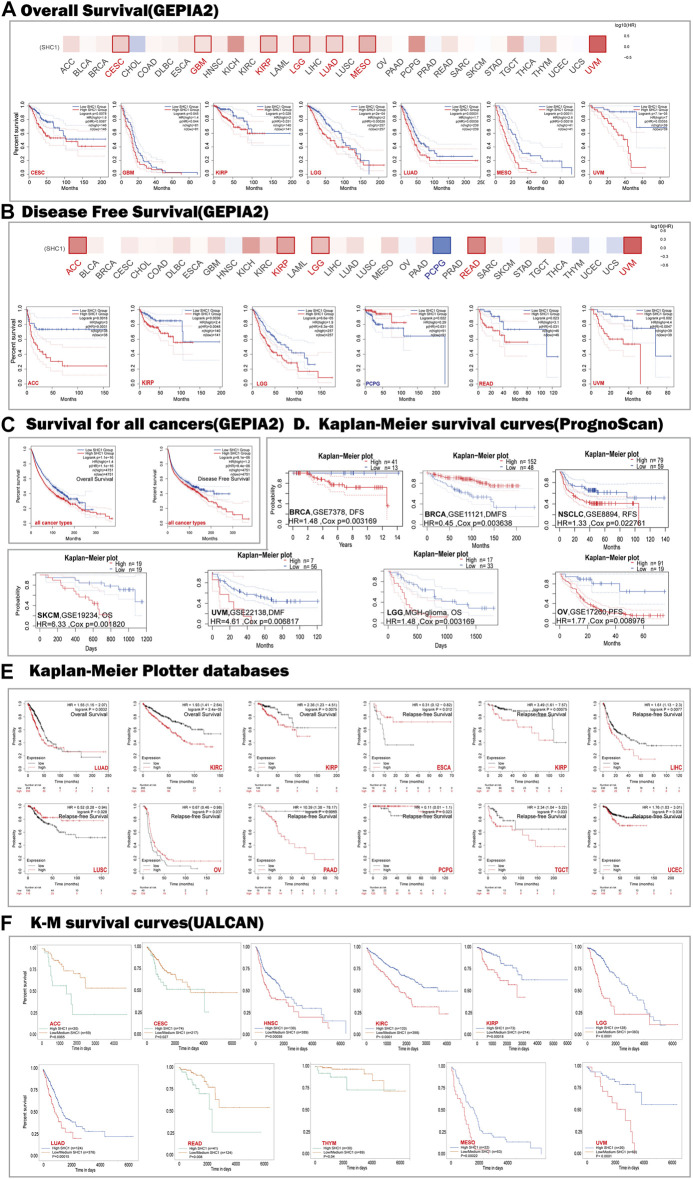
Correlation between SHC1 expression and survival prognosis values in various cancers. The overall survival (OS) **(A)** disease-free survival (DFS) **(B)** of different tumors, overall survival, and disease-free survival of all cancer types **(C)** in TCGA determined by GEPIA2. The survival map and Kaplan-Meier curves with positive results are shown. Relationship between SHC1 expression and patient prognosis of different cancer datasets obtained using PrognoScan **(D)**. Kaplan-Meier survival curves showing the survival of patients with high and low expression of SHC1 in Kaplan-Meier Plotter **(E)**. Kaplan-Meier survival curves showing the comparison between the high and low expression of SHC1 in UALCAN **(F)**. DFS, disease-free survival; DMFS, distant metastasis-free survival; RFS, relapse-free survival; OS, overall survival; PFS, progression-free survival.

We dichotomized the cancer cohort into high and low groups based on the median SHC1 mRNA-expression. Next, we evaluated the relationship between SHC1 expression and patient clinical outcomes across different tumors using the Kaplan-Meier analysis. It was realized that high levels of SHC1 expression correlated with poor OS in patients with CESC (*p* = 0.0078), GBM (*p* = 0.045), KIRP (*p* = 0.028), LGG (*P* = 2e−04), LUAD (*p* = 0.00031), MESO (*p* = 0.00011), and UVM (*p* = 7.1e−05) from the TCGA dataset (Figure 2A). Furthermore, high SHC1 expression was significantly correlated with poor disease-free survival (DFS) in patients with ACC (*p* = 0.0016), KIRP (*p* = 0.0036), LGG (*p* = 6.6e−05), READ (*p* = 0.023), and UVM (*p* = 0.002). Conversely, it was found that high SHC1 expression was linked to a greater DFS in PCPG (*p* = 0.022). The overall effect of SHC1 expression on the prognosis of all cancers was demonstrated in Figure 2C. We found that high expression of SHC1 was generally strongly correlated with a worse prognosis in cancers (OS: total number = 9502, HR = 1.4, logrank *p* = 1.1e−16; DFS: total number = 9502, HR = 1.2, logrank *p* = 8.4E−06).

The relationships between SHC1 expression and the outlook of each cancer type was determined using the PrognoScan web-based tool. Notably, high SHC1 expression has negative effects on six cancers, including BRCA (DFS: total number = 54, HR = 1.48, Cox *p* = 0.003169), NSCLC (RFS: total number = 138, HR = 1.33, Cox *p* = 0.022761), SKCM(OS: total number = 38, HR = 6.33, Cox *p* = 0.001820), UVM (distant metastasis-free survival (DMFS): total number = 63, HR = 4.61, Cox *p* = 0.006817), LGG (OS: total number = 50, HR = 1.77, Cox *p* = 0.003169), and OV(progression-free survival (PFS): total number = 110, HR = 1.77, Cox *p* = 0.008976). In contrast, high SHC1 expression had a protective impact on the BRCA (DMFS: total number = 200, HR = 0.45, Cox *p* = 0.003648) (Figure 2D).

The SHC1-related overall survival and relapse free survival were then evaluated using the Kaplan-Meier Plotter database. Similarly, SHC1 overexpression was found to be a prognostic risk factor for LUAD patients (OS: HR = 1.55, 95% CI from 1.15 to 2.07, logrank *p* = 0.0032), KIRC (OS: HR = 1.93, 95% CI from 1.41 to 2.64, logrank *p* = 2.4e−05), KIRP (OS: HR = 2.36, 95% CI from 1.23 to 4.51, logrank *p* = 0.0075; RFS, HR = 3.49, 95% CI from 1.61 to 7.57, logrank *p* = 0.00075), LIHC (RFS, HR = 1.61, 95% CI from 1.13 to 2.3, logrank *p* = 0.0077), PAAD (RFS, HR = 10.39, 95% CI from 1.38 to 78.17, logrank *p* = 0.0053), TGCT (RFS, HR = 2.34, 95% CI from 1.04 to 5.22, logrank *p* = 0.033), and UCEC (RFS, HR = 1.76, 95% CI from 1.03 to 3.01, logrank *p* = 0.038). Conversely, low expression levels of SHC1 were found to be related to poor RFS for ESCA (HR = 0.31, 95% CI from 0.12 to 0.82 logrank *p* = 0.012), LUSC (HR = 0.52, 95% CI from 0.28 to 0.94, logrank *p* = 0.028), OV (HR = 0.67, 95% CI from 0.46 to 0.98, logrank *p* = 0.037), PCPG (HR = 0.11, 95% CI from 0.01 to 1.1, logrank *p* = 0.023) (Figure 2E).

As expected, UALCAN data demonstrated that patients with high SHC1 levels had were significantly related to a poor survival rate for ACC (*p* = 0.0065), CESC (*p* = 0.027), HNSC (*p* = 0.00058), KIRC (*p* < 0.0001), KIRP(*p* = 0.00018), LGG (*p* < 0.0001), LUAD (*p* = 0.00015), READ (*p* = 0.008), THYM (*p* = 0.04), MESO (*p* = 0.00022), and UVM (*p* < 0.0001) (Figure 2F).

The relationship between SHC1 expression and OS, PFS, DFI, and DSS in 33 cancer types were shown by the forest plots (Supplementary Figure S1). The expression of SHC1 was linked to patient survival and appeared to be a risk factor in 12 types of cancer, including ACC, CESC, HNSC, KICH, KIRC, KIRP, LAML, LGG, LIHC, LUAD, MESO, and UVM (Supplementary Figure S1A). Similarly, we found that the abnormal expression of SHC1 was associated with poor PFS in 11 types of cancer, including CESC, HNSC, KICH, KIRC, KIRP, LGG, LIHC, LUAD, MESO, THCA and UVM (Supplementary Figure S1B). Notably, SHC1 expression was significantly correlated with DFS in various types of cancer, including BRCA, CESC, KICH, KIRC, KIRP, LGG, LIHC, LUAD, READ, UCEC, and UVM (Supplementary Figure S1C). Moreover, SHC1 expression was found to affect DSS in patients of seven cancer types, including ACC, BRCA, KIRP, LIHC, MESO, PAAD, and UCEC (Supplementary Figure S1D).

These results indicated that SHC1 expression is significantly correlated with the prognosis of patients with certain cancer types, particularly CESC, KICH, KIRC, KIRP, LGG, LIHC, LUAD, and UVM.

### Genetic Alteration Analysis in Cancers

**FIGURE 3:**
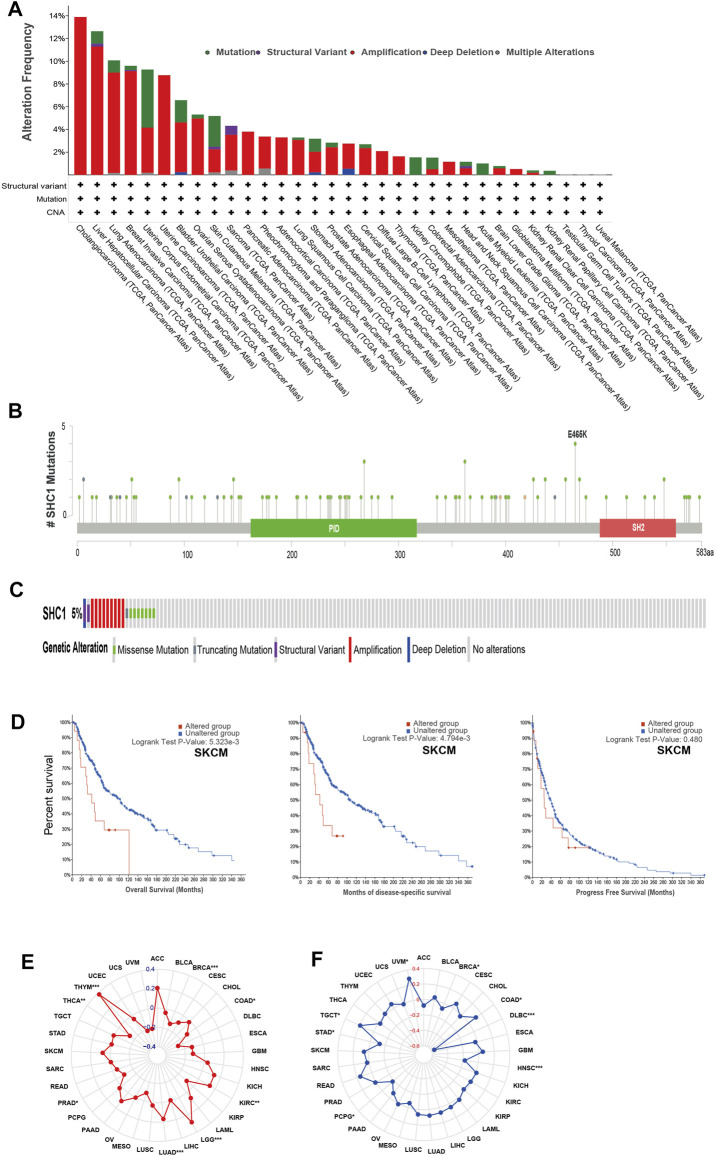
The landscape of mutation signatures of SHC1 in TCGA. The relative frequency of each mutation type **(A)** and mutation site **(B)**. **(C)** OncoPrint visualization of SHC1 alterations. **(D)** Kaplan–Meier plots showing the comparison of OS, DSS, PFS in cases with/without SHC1 gene alterations in SKCM developed using the cBioPortal tool. The radargram of TMB **(E)**, MSI **(F)**, and SHC1 expressions in various cancers. (Spearman Correlation test, *p* values < 0.05 was considered statistically significant, *p < 0.05, **p < 0.01, and ***p < 0.001).

We used the cBioPortal database to estimate the genetic alteration status of SHC1, including mutations, structural variants, amplifications, deep deletions, and multiple alterations. The highest mutation frequency of SHC1 (>13%) was found in patients with CHOL, with “Amplification” as the predominant type (Figure 3A). The “mutation,” as the primary type, was found in UCEC patients. It is worth noting that copy number amplification of SHC1 was found in cases of CHOL, UCEC, PAAD, and ACC with genetic mutation (2–8% frequency). Additionally, based on the sites and number of cases of the SHC1 genetic alteration (Figure 3B), we noted that missense mutation of SHC1 was the predominant form in various cancer types. Specifically, the percentage change in genetic variations of SHC1 across all TCGA tumors was 5% (Figure 3C). Furthermore, we estimated the potential relationships between SHC1genetic changes and clinical outcomes in various cancers. It was found that SKCM cases with altered SHC1 had a markedly worse prognosis in OS (*p* = 5.323e−3) and DSS (*p* = 4.794e−3), but were not remarkably associated with PFS (*p* = 0.480, Figure 3D). Currently, numerous studies have revealed that TMB and MSI may act as a potential predictive markers of cancer immunotherapeutic response and prognosis (Davis et al., 2017; Yarchoan et al., 2017; Hellmann et al., 2018; Zhao et al., 2019). Therefore, we assessed the correlation between SHC1 expression and TMB/MSI in pan-cancer. It was discovered that SHC1 expression was negatively correlated with TMB in BRCA (*p* < 0.001), COAD (*p* < 0.05), PRAD (*p* < 0.05), THCA (*p* < 0.01), and THYM (*p* < 0.001), but positively correlated with TMB in KIRC (*p* <0.01), and LGG (*p* < 0.001) (Figure 3E). The expression of SHC1 was negatively correlated with MSI in BRCA (*p* < 0.01), DLBC (*p* < 0.001), PCPG (*p* < 0.05), and STAD (*p* < 0.05), but positively correlated with MSI in COAD (*p* < 0.05), HNSC (*p* < 0.001), TGCT (*p* < 0.05), and UVM (*p* < 0.05) (Figure 3F).

### The Relationship Between SHC1 Expression Levels and MMR, DNMT, and m6A RNA Modification Regulator Genes in Human Pan-Cancer

**FIGURE 4:**
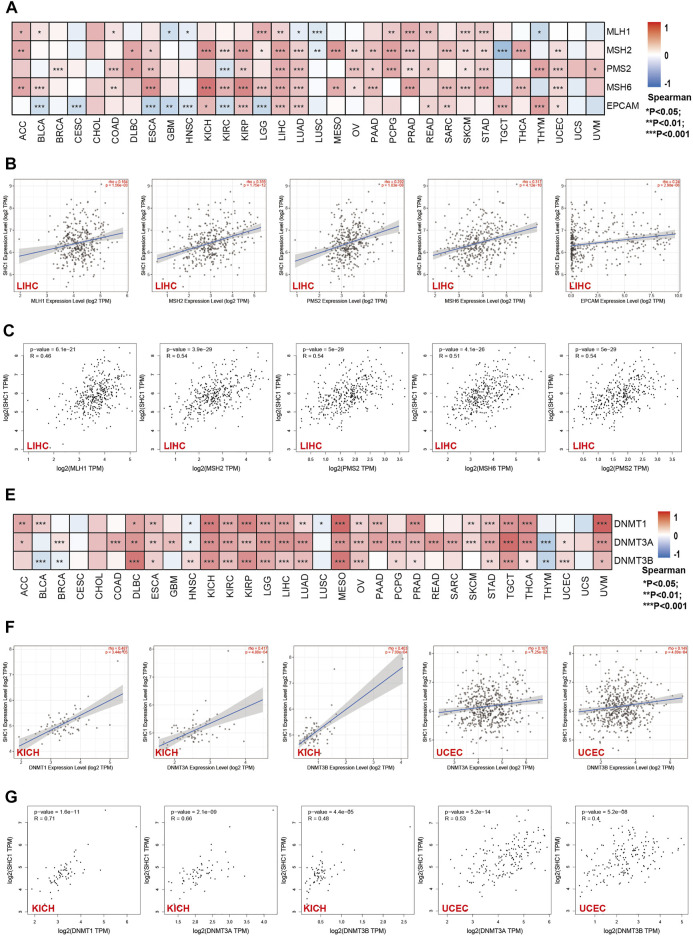
Correlation between SHC1 expression and the expression of MMRs and DNMTs genes in various cancers. **(A)** Heatmap showing the Spearman correlation analysis of SHC1 expression and the levels of five MMR genes (MLH1, MSH2, MSH6, PMS2, and EPCAM) in pan-cancer. Correlation between SHC1 expression and expression of MMR genes in LIHC plotted using TIMER2.0 **(B)** and confirmed *via* GEPIA2 **(C)**. Heatmap showing the Spearman correlation between the SHC1 expression and of the expression of three DNA methyltransferase (DNMT1, DNMT3A, and DNMT3B) genes in pan-cancer **(E)**. Correlation between SHC1 expression and expression of DNA methyltransferase genes in LIHC and UCEC plotted using TIMER2.0 **(F)** and confirmed *via* GEPIA2 **(G)**. (*p < 0.05, **p < 0.01, and ***p < 0.001).

**FIGURE 5:**
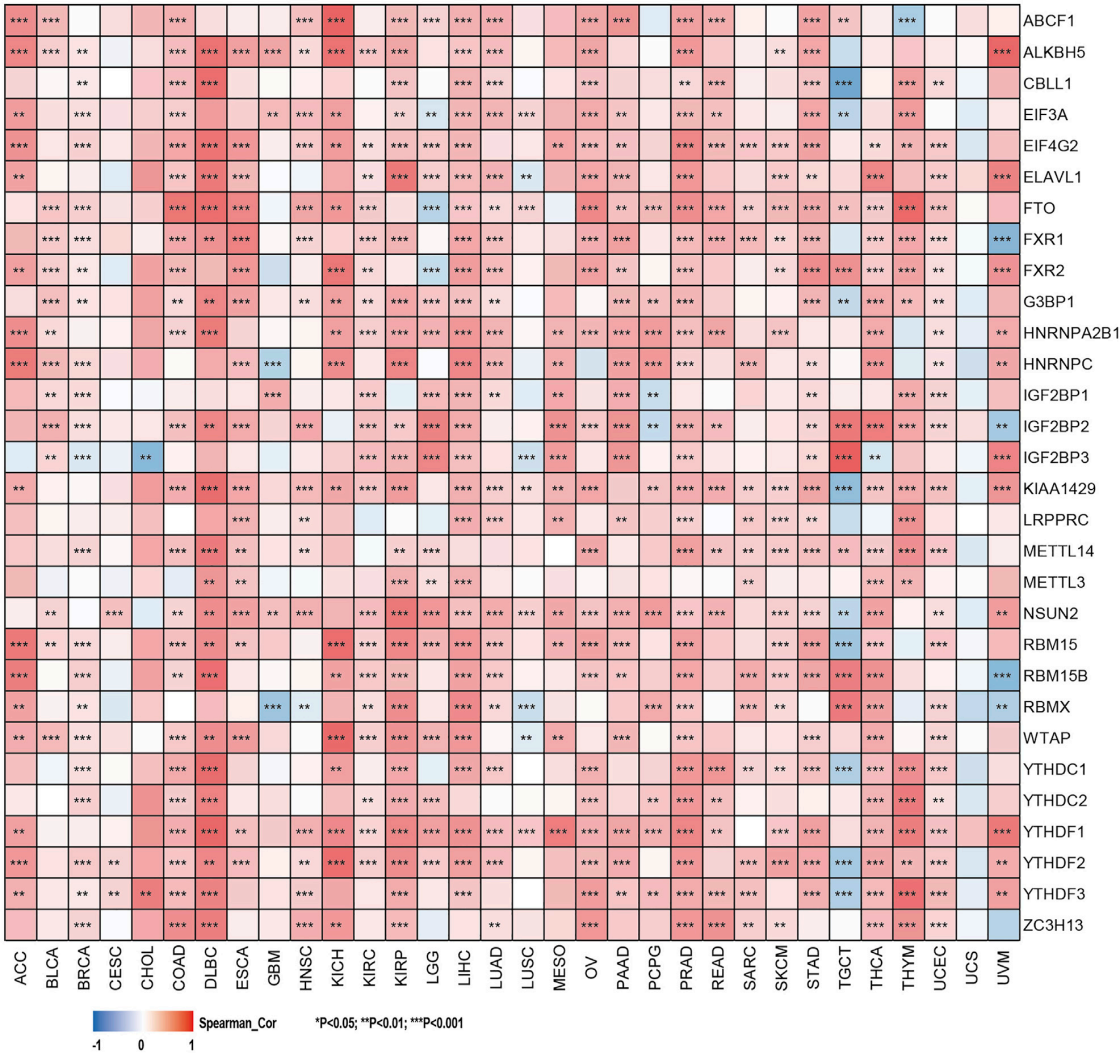
Correlation of SHC1 gene expression levels with the most common m6A RNA modification regulator levels in pan-cancer.

The relationship between the expression levels of SHC1 and DNA methyltransferases and MMR gene levels were assessed using the TIMER2.0 and the GEPIA2.0 database was used to verify the role of SHC1 in tumorigenesis. It was revealed that SHC1 and five MMR genes (MLH1, MSH2, MSH6, PMS2, and EPCAM) were positively correlated in human cancers (Figure 4A), especially in the LIHC (Figures 4B,C). Evidently, there was a close relationship between SHC1 expression and the levels of DNMT1, DNMT3A, and DNMT3B in DLBC, KICH, KIRC, KIRP, LGG, LIHC, LUAD, MESO, TGCT, and UVM (Figures 4E–G). Subsequently, the correlation between SHC1 expression and that of 30 common m6A RNA modification regulators with different types of cancer was evaluated. Interestingly, SHC1 expression was correlated with more than 20 m6A RNA modification regulators, such as FTO, KIAA1429, and YTHDF2 in BRCA, COAD, DLBC, KIRP, LIHC, LUAD, OV, PRAD, PRAD, STAD, THCA, and UCEC (Figure 5). These results strongly indicate that SHC1 may mediate tumorigenesis by regulating DNA damage, DNA methylation, or RNA methylation.

### SHC1 Protein Phosphorylation Analysis in Various Tumors

**FIGURE 6:**
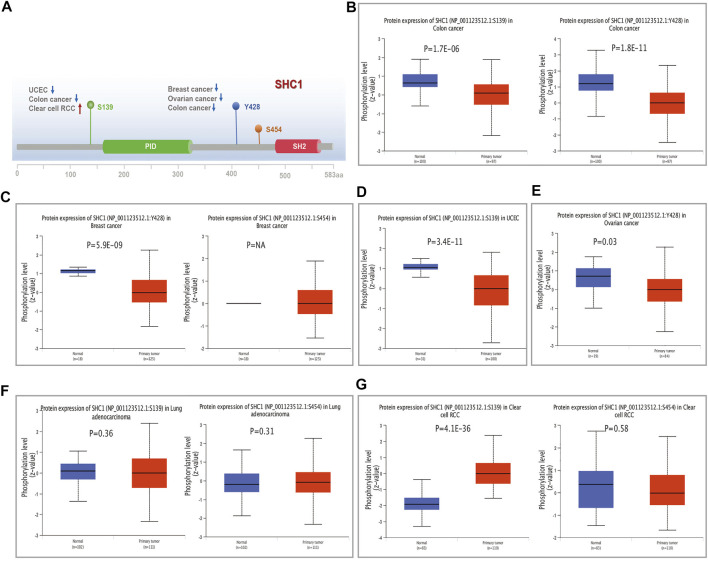
The distribution of SHC1 protein phosphorylation in different tumors. The phosphoprotein levels presented as a schematic diagram of SHC1 protein **(A)**. The phosphoprotein levels of SHC1 in normal tissue and different cancer tissues, including colon cancer **(B)**, breast cancer **(C)**, UCEC **(D)**, ovarian cancer **(E)**, LUAD **(F)**, and clear cell RCC **(G)**.

We examined the level of SHC1 phosphoprotein (NP_001123512.1, S139, Y428, and S454 sites) between normal and six types of tumor tissues (breast cancer, UCEC, ovarian cancer, lung adenocarcinoma, colon cancer, and clear cell RCC) based on the CPTAC dataset, using the UALCAN web resource. Figure 6A shows the SHC1 phosphorylated sites and the significant differences among the cancer types. The S139 locus of SHC1 exhibited a lower phosphorylation level in colon cancer (Figure 6B, *p* = 1.7e−06) and UCEC (Figure 6D, *p* = 3.4e−11) and higher phosphorylation level in clear cell RCC (Figure 6G, *p* = 4.1e−36) compared to normal tissues. The Y428 locus of SHC1 exhibited a lower phosphorylation level in breast cancer (Figure 6C, *p* = 5.9e−09), ovarian cancer (Figure 6E, *p* = 0.03) and colon cancer (Figure 6B, *p* = 1.8e−11) compared to normal tissues. However, no phosphorylated cancer types were found at the S454 locus of SHC1 (Figures 6A–G). This finding merits further molecular analysis to investigate the potential role of S139 and Y428 phosphorylation in tumorigenesis.

### SHC1-Related Immune Infiltration, Immune Checkpoint, and TAMs

**FIGURE 7:**
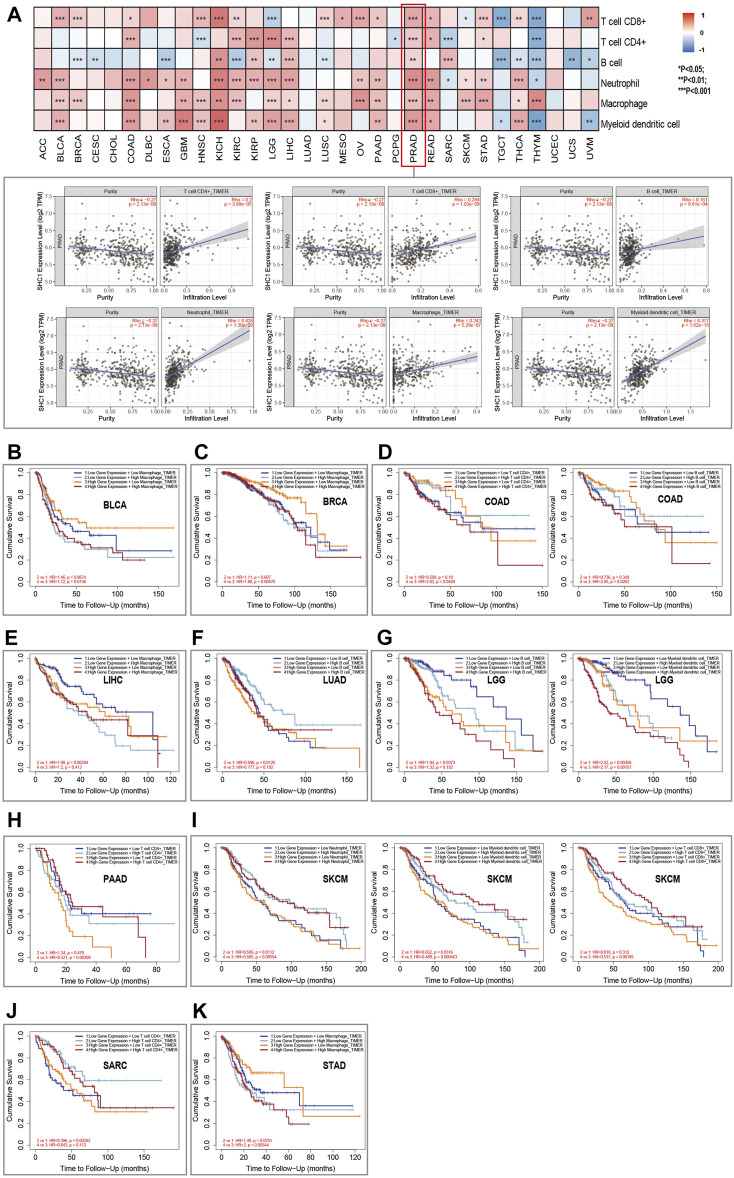
Relationship between SHC1 expression and immune infiltration in various tumors. **(A)** Correlation of SHC1 expression with immune infiltration levels of CD4^+^ T cells, CD8^+^ T cells, B cells, neutrophil cells, macrophage cells, and myeloid dendritic cells. Survival analysis of SHC1 expression level and Macrophage infiltration level in BLCA **(B)**, BRCA **(C)**. Survival analysis of SHC1 expression level and infiltration levels of CD4^+^ T cell or B cell in COAD **(D)**. Survival analysis of SHC1 expression level and Macrophage infiltration level in LIHC **(E)**. Survival analysis of SHC1 expression level and B cell infiltration level in LUAD **(F)**. Survival analysis of SHC1 expression level and infiltration levels of B cell and myeloid dendritic cell in LGG **(G)**. Survival analysis of SHC1 expression level and CD4^+^ T infiltration level in PAAD **(H)**. Survival analysis of SHC1 expression level and infiltration levels of Neutrophil, myeloid dendritic cell, and CD8^+^ T cell in SKCM **(I)**. Survival analysis of SHC1 expression level and CD4^+^ T cell infiltration level in SARC **(J)**. Survival analysis of SHC1 expression level and Macrophage infiltration level in STAD **(K)**. *p* < 0.05 was considered statistically significant.

TIMER2.0 database was used to assess the correlation between SHC1 expression and the immune cell infiltration in pan-cancer. Results indicated that the expression of Tumor-related SHC1 was significantly correlated with the tumor-infiltration of CD8^+^ T cells, CD4^+^ T cells, B cells, neutrophils, macrophages, and myeloid dendritic cells in 19, 12, 16, 20, 18, and 17 cancers, respectively (Figure 7A). Furthermore, SHC1 expression levels were positively correlated with immune cells in PRAD. The scatterplot data are presented in Figure 7A. However, SHC1 expression was not significantly correlated with immune cells in CHOL, LUAD, and UCEC.

The Kaplan–Meier survival curves were used to assess the impact of aberrant SHC1 expression and immune cell infiltration on OS. Results indicated that BLCA, BRCA, and STAD patients with high expression of SHC1 and macrophage infiltration had poor OS (Figures 6B,C,K). COAD patients with high SHC1 expression, CD4^+^ T infiltration, and B cells infiltration also showed poor OS (Figure 7D). LIHC patients with low SHC1 expression level and infiltration level of macrophage had a relatively good OS (Figure 7E). In LUAD, the low SHC1 expression and high B cell infiltration showed a good OS (Figure 7F). Meanwhile, LGG patients with low SHC1 expression, B cell infiltration level, and myeloid dendritic cell infiltration level had a relatively good OS. In contrast, patients with high SHC1 expression and high myeloid dendritic cell infiltration level had poor OS (Figure 7G). PAAD patients with high infiltration levels of CD4^+^ T cell and high SHC1 expression level showed good OS (Figure 7D). Similarly, SARC patients with high infiltration levels of CD4^+^ T cell and low SHC1 expression level also had good OS (Figure 7J). Besides, SKCM patients with high SHC1 expression and low infiltration levels of neutrophils, myeloid dendritic cells, and CD8^+^ T cells had a relatively poor OS. However, patients with low SHC1 expression and high neutrophil infiltration levels had good OS (Figure 7I).

**FIGURE 8:**
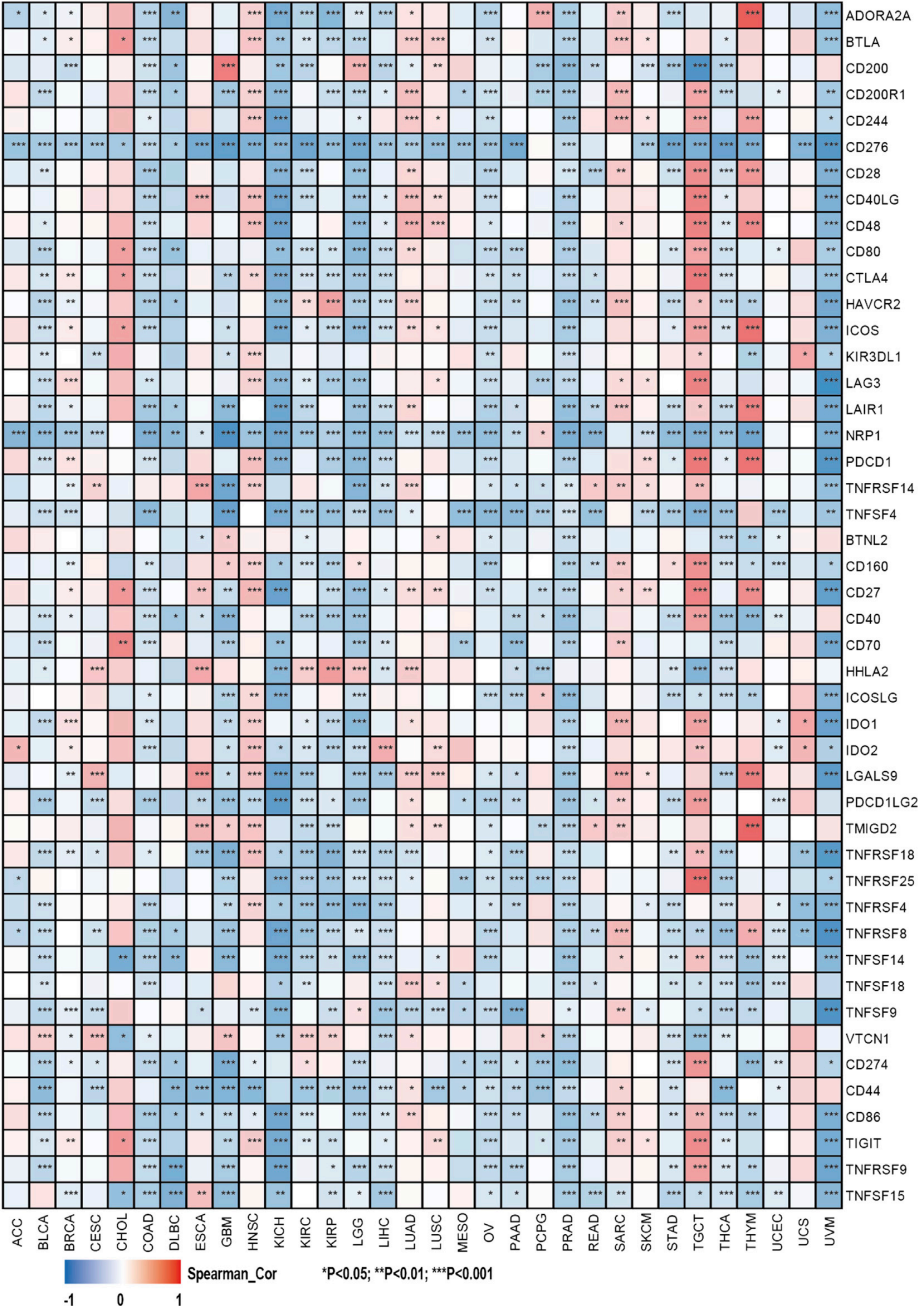
Correlation analysis of SHC1 expression levels with the most common immune checkpoint gene levels in pan-cancer.

This study also investigated the association between the expression of over 40 common immune checkpoint genes from the previous study and the SHC1 expression (Liu et al., 2020). Interestingly, CD276, NRP1, and TNFSF4 expressions were significantly negatively correlated with SHC1 in various cancers. SHC1 expression was also associated with more than 30 immune checkpoints in COAD, KICH, LGG, PRAD, THCA, TGCT, and UVM. Additionally, SHC1 expression and immune checkpoint markers were positively correlated in HNSC, LUAD, SARC, and TGCT patients. Conversely, they were negatively correlated in COAD, KICH, LGG, PRAD, THCA, and UVM (Figure 8).

**FIGURE 9:**
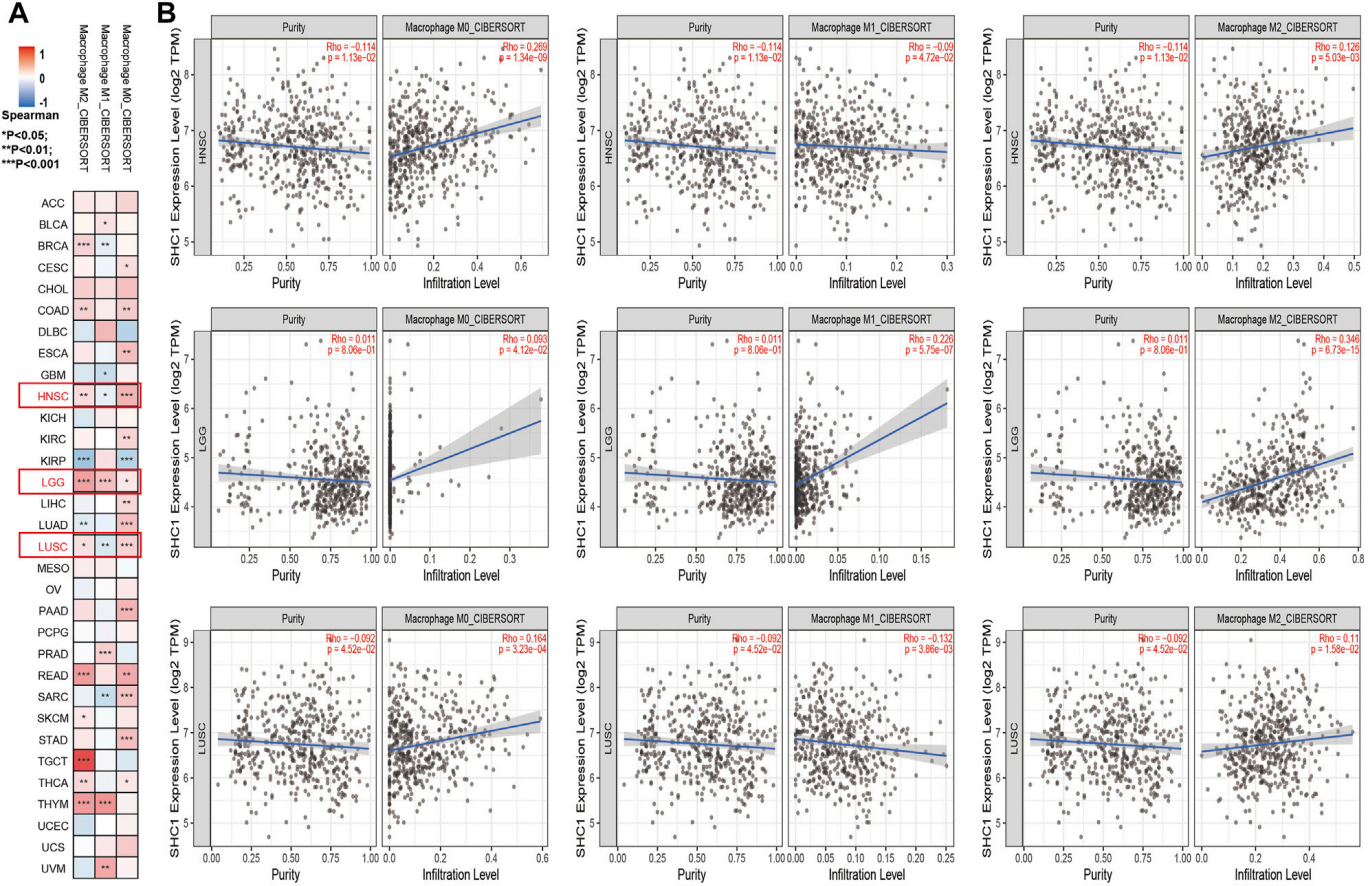
Correlation analysis between SHC1 expression and tumor-associated macrophages (TAMs). **(A)** CIBERSORT algorithms were used to explore the potential correlation between the expression level of the SHC1 gene and the infiltration level of cancer-associated fibroblasts across all types of cancer in TCGA. **(B)** The correlation analysis chart of HNSC, LGG, LUSC are given.

The effect of SHC1 on the tumor-associated macrophage (TAM) infiltration was also assessed. M0, M1, and M2 macrophage infiltration levels were significantly correlated with the SHC1 expression in most tumors, especially in HNSC, LGG, and LUSC (Figure 9). TIMER2.0 and GEPIA2.0 were used to assess the relationship between SHC1 expression and markers of TAMs, M1, and M2 macrophages (Tables 2, 3). Interestingly, TAMs (CCL2, CD86, and IL10), M1 [INOS(NOS2), IRF5, and COX2(PTGS2)] were significantly correlated with SHC1 expression in the three cancers (Table 2). However, M2 (CD163, VSIG4, and MS4A4A) was significantly correlated with SHC1 expression in only HNSC and LGG. Taken together, these results suggest that SHC1 plays an essential role in tumor immunity.

**TABLE 2:** Correlations between SHC1 and gene markers of immune cells in TIMER.

Description	Gene markers	HNSC (*n* = 522)				LGG (*n* = 516)				LUSC (*n* = 501)			
		None		Purity		None		Purity		None		Purity	
		Cor	*p*	Cor	*p*	Cor	*p*	Cor	*p*	Cor	*p*	Cor	*p*
TAM	CCL2	−0.008	0.8530	−0.020	0.6511	0.345	***	0.350	***	0.029	0.5106	−0.011	0.8155
	CD68	0.226	***	0.211	***	0.337	***	0.340	***	0.139	**	0.105	*
	IL10	0.080	0.0684	0.060	0.1829	0.311	***	0.301	***	0.020	0.6624	−0.026	0.5740
M1	INOS(NOS2)	−0.160	***	−0.140	*	−0.031	0.4860	−0.008	0.8652	−0.242	***	−0.227	***
	IRF5	−0.147	***	−0.143	*	0.317	***	0.340	***	0.192	***	0.197	***
	COX2(PTGS2)	0.099	*	0.125	*	0.099	*	0.099	*	0.144	*	0.127	**
M2	CD163	0.150	***	0.123	*	0.452	***	0.431	***	0.083	0.0641	0.055	0.2285
	VSIG4	0.103	*	0.073	0.1035	0.197	***	0.182	***	0.000	0.9978	−0.036	0.4314
	MS4A4A	0.089	*	0.066	0.1426	0.330	***	0.311	***	−0.004	0.9352	−0.041	0.3766

TAM, tumor-associated-macrophage; None, correlation without adjustment; Purity, correlation adjusted by for tumor purity; Cor, R value of Spearman’s correlation. *p < 0.05; **p < 0.01; ***p < 0.001.

**TABLE 3:** Correlations between SHC1 and gene markers of immune cells in GEPIA.

Description	Gene markers	HNSC (*n* = 522)				LGG (*n* = 516)		LUSC (*n* = 501)			
		Tumor		Normal		Tumor		Tumor		Normal	
		Cor	*p*	Cor	*p*	Cor	*p*	Cor	*p*	Cor	*p*
TAM	CCL2	0.11	*	0.33	*	0.33	***	0.088	0.052	0.052	0.055
	CD68	0.45	***	0.23	0.1	0.35	***	0.23	0.1	0.23	0.1
	IL10	0.26	***	0.32	*	0.33	***	0.11	*	0.32	*
M1	INOS(NOS2)	0.4	**	−0.041	0.35	0.074	0.094	−0.21	***	0.4	***
	IRF5	−0.023	0.6	0.56	***	0.31	***	0.21	***	0.061	0.67
	COX2(PTGS2)	0.23	***	0.43	**	0.22	***	0.19	***	0.39	***
M2	CD163	0.15	***	0.32	*	0.42	***	0.13	**	0.31	*
	VSIG4	0.18	***	0.38	*	0.23	***	0.1	*	−0.11	0.47
	MS4A4A	0.14	***	0.34	*	0.34	***	0.088	0.054	0.088	0.054

TAM, tumor-associated- macrophage; Tumor, correlation analysis in tumor tissue of TCGA; Normal, correlation analysis in normal tissue of TCGA; Cor, R value of Spearman’s correlation. *p < 0.05; **p < 0.01; ***p < 0.001.

### Diagnostic Value of SHC1 for Pan-Cancer

**FIGURE 10:**
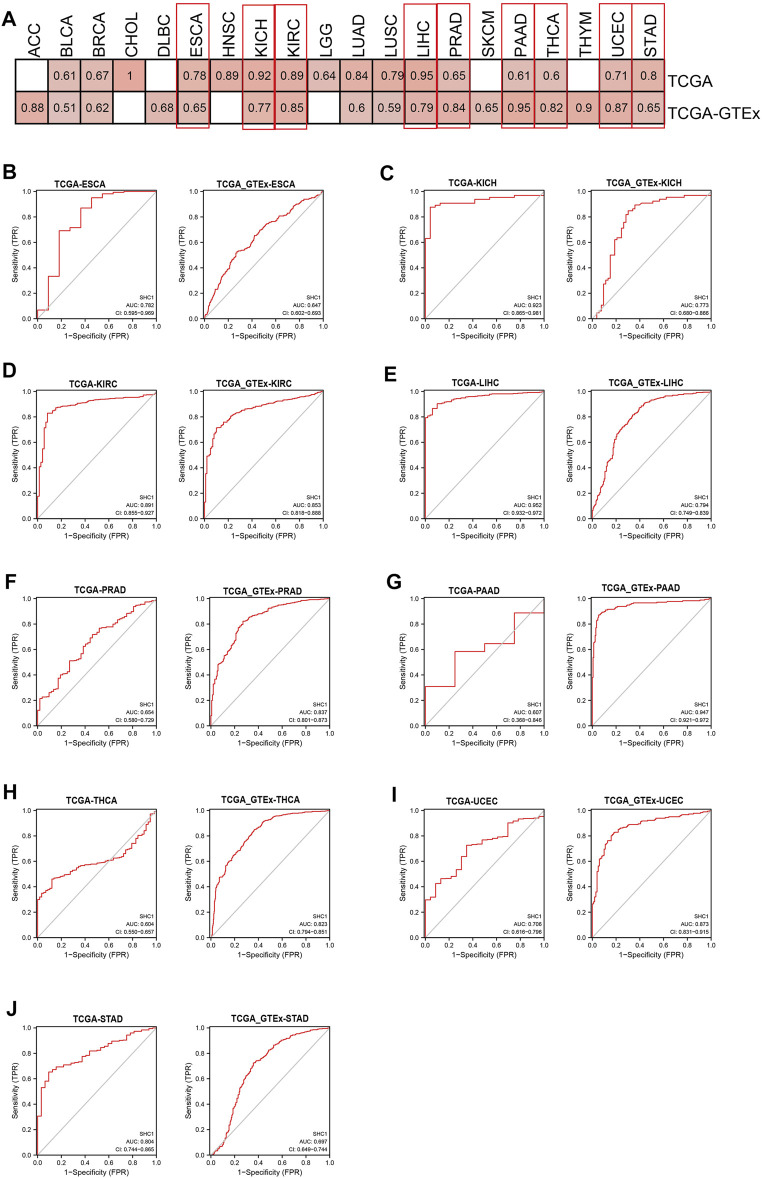
Diagnostic efficiency of SHC1 gene in different tumors. **(A)** The heatmap shows the AUC values of SHC1 gene expression level in the diagnosis of various cancers. The ROC curve of SHC1 gene in diagnosis of ESCA **(B)**, KICH **(C)**, KIRC **(D)**, LIHC **(E)**, PRAD **(F)**, PAAD **(G)**, THCA **(H)**, UCEC **(I)**, and STAD **(J)** using the TCGA and TCGA-GTEx dataset.

The diagnostic value of SHC1 in various cancers of TCGA was investigated, then confirmed using the normal tissue of the GTEx dataset (controls) (Table 4). Heat map was used to express the AUC values in most cancers (Figure 10A). SHC1 had a strong diagnostic efficacy in various tumors, especially ESCA, KICH, KIRC, LIHC, PRAD, PAAD, THCA, UCEC, and STAD. ROC curve (TCGA dataset) showed that SHC1 expression levels had strong (AUC>0.9) diagnostic potency in CHOL [AUC = 1.00 (1.000–1.000)], KICH [AUC = 0.923 (0.865–0.981)], and LIHC [AUC = 0.952 (0.932–0.972)], and moderate (0.7 < AUC<0.9) diagnostic potency in ESCA [AUC = 0.782 (0.595–0.969)], HNSC [AUC = 0.891 (0.855–0.927)], KIRC [AUC = 0.891 (0.855–0.927)], LUAD [AUC = 0.842 (0.804–0.879)], LUSC [AUC = 0.795 (0.744–0.845)], UCEC [AUC = 0.706 (0.616–0.796)], and STAD [AUC = 0.804 (0.744–0.865)]. For the normal tissue of the GTEx dataset, SHC1 expression levels showed strong diagnostic potency only in PAAD [AUC = 0.947 (0.921–0.972)]. Moderate diagnostic potency was observed in ACC [AUC = 0.884 (0.826–0.942)], KICH [AUC = 0.773 (0.680–0.866)], KIRC [AUC = 0.853 (0.855–0.927)], LIHC [AUC = 794 (0.749–0.839)], PRAD [AUC = 0.837 (0.801–0.873)], THCA [AUC = 0.823 (0.794–0.851)], and UCEC [AUC = 0.873 (0.831–0.915)]. These data imply that SHC1 has potential diagnostic value in ESCA, KICH, KIRC, LIHC, PRAD, PAAD, THCA, UCEC, and STAD.

**TABLE 4:** The diagnostic value of SHC1 for pan-cancer.

Cancer	Dada set	Tumor (*n*)	Normal (*n*)	AUC	Confidence interval (CI)	Cut-off	Sensit ivity	Specif icity	PPV	NPV	Youden index
ACC	TCGA-GTEx	77	128	0.884	0.826–0.942	6.061	0.857	0.867	0.795	0.91	1.724
BLCA	TCGA	414	19	0.605	0.522–0.687	6.717	0.425	0.895	0.989	0.067	1.32
	TCGA-GTEx	407	28	0.512	0.425–0.600	5.698	0.371	0.786	0.962	0.079	1.157
BRCA	TCGA	1083	111	0.666	0.628–0.705	6.964	0.438	0.91	0.979	0.142	1.348
	TCGA-GTEx	1099	292	0.621	0.587–0.655	6.246	0.357	0.846	0.897	0.259	1.203
CHOL	TCGA	36	9	1	1.000–1.000	5.618	1	1	1	1	2
DLBC	TCGA-GTEx	47	444	0.682	0.602–0.763	4.883	0.447	0.854	0.244	0.936	1.3
ESCA	TCGA	162	11	0.782	0.595–0.969	6.24	0.691	0.818	0.982	0.153	1.51
	TCGA-GTEx	182	666	0.647	0.602–0.693	6.368	0.527	0.727	0.345	0.849	1.254
HNSC	TCGA	502	44	0.891	0.855–0.927	6.381	0.805	0.909	0.99	0.29	1.714
KICH	TCGA	65	24	0.923	0.865–0.981	5.39	0.877	0.958	0.983	0.742	1.835
	TCGA-GTEx	66	53	0.773	0.680–0.866	5.122	0.848	0.698	0.778	0.787	1.547
KIRC	TCGA	539	72	0.891	0.855–0.927	6.145	0.829	0.917	0.987	0.418	1.746
	TCGA-GTEx	531	100	0.853	0.818–0.888	6.026	0.714	0.9	0.974	0.372	1.614
LGG	TCGA-GTEx	523	1152	0.637	0.611–0.663	3.715	0.881	0.391	0.397	0.879	1.273
LUAD	TCGA	535	59	0.842	0.804–0.879	6.57	0.693	0.915	0.987	0.248	1.609
	TCGA-GTEx	515	347	0.602	0.563–0.640	6.575	0.621	0.556	0.675	0.497	1.178
LUSC	TCGA	502	49	0.795	0.744–0.845	6.67	0.681	0.816	0.974	0.2	1.498
	TCGA-GTEx	498	338	0.565	0.526–0.604	6.101	0.325	0.846	0.757	0.46	1.171
LIHC	TCGA	374	50	0.952	0.932–0.972	5.557	0.866	0.94	0.991	0.485	1.806
	TCGA-GTEx	371	160	0.794	0.749–0.839	5.106	0.871	0.606	0.837	0.669	1.477
PRAD	TCGA	499	52	0.654	0.580–0.729	6.313	0.717	0.538	0.937	0.166	1.256
	TCGA-GTEx	496	152	0.837	0.801–0.873	5.935	0.823	0.73	0.909	0.558	1.553
SKCM	TCGA-GTEx	469	813	0.646	0.615–0.676	6.243	0.812	0.571	0.522	0.841	1.383
PAAD	TCGA-GTEx	179	171	0.947	0.921–0.972	5.542	0.872	0.947	0.945	0.876	1.819
THCA	TCGA	510	58	0.604	0.550–0.657	6.495	0.461	0.879	0.971	0.156	1.34
	TCGA-GTEx	512	338	0.823	0.794–0.851	6.341	0.848	0.636	0.779	0.734	1.484
THYM	TCGA-GTEx	119	446	0.895	0.863–0.927	5.039	0.739	0.895	0.652	0.928	1.634
UCEC	TCGA	552	23	0.706	0.616–0.796	6.313	0.726	0.652	0.98	0.09	1.379
	TCGA-GTEx	181	101	0.873	0.831–0.915	6.065	0.823	0.822	0.892	0.722	1.645
STAD	TCGA	375	32	0.804	0.744–0.865	5.969	0.653	0.906	0.988	0.182	1.56
	TCGA-GTEx	414	210	0.697	0.649–0.744	5.679	0.725	0.643	0.8	0.542	1.367

AUC, Area under the curve; PPV, positive predictive value; NPV, negative predictive value.

### Enrichment Analysis of SHC1-Related Partners

**FIGURE 11:**
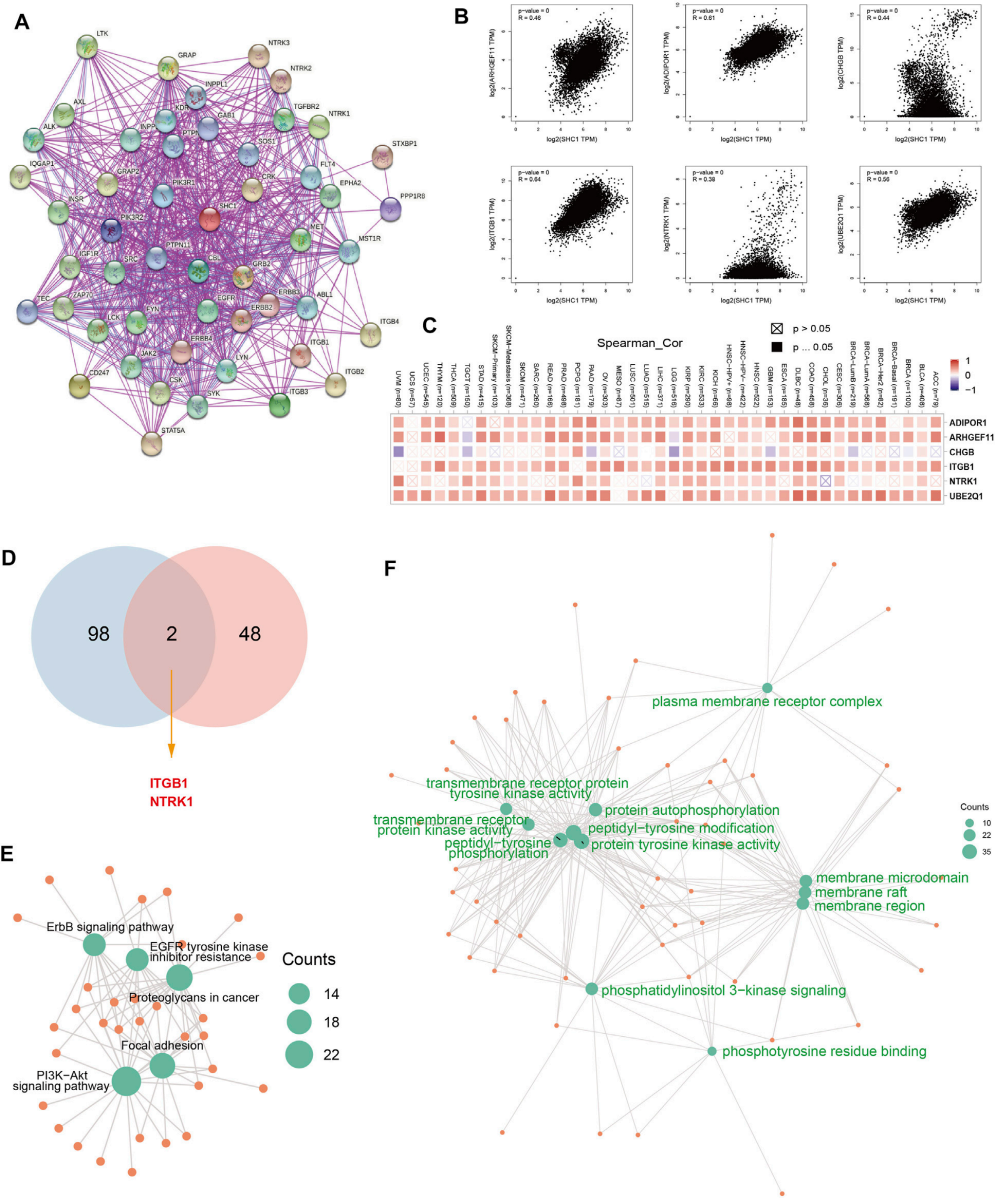
SHC1-related gene enrichment analysis. **(A)** The experimentally determined SHC1-binding proteins was obtained using the web tool STRING. **(B)** The top 100 SHC1 Similar Genes, were also gained using the GEPIA2 approach and the expression correlation between SHC1 and the top five selected targeting genes, including ADIPOR1, ARHGEF11, CHGB, ITGB1and NTRK1 were analyzed. **(C)** The heatmap displayed the correlation between SHC1 expression and the expression profiles of five selected targeting genes across the analyzed cancer types. **(D)** Venn diagram was conducted to display the intersection of the SHC1-binding and correlated genes. Based on the SHC1-binding and interacted genes, KEGG pathway analysis **(E)** and GO analysis **(F)** was performed. CC, cellular component; BP, biological process; MF, molecular function.

The SHC1-binding proteins and the SHC1 expression-correlated genes were screened using the STRING tool for enrichment analyses to further determine the underlying molecular mechanisms of the SHC1 in tumorigenesis. A total of 50 SHC1-binding proteins were identified. The interaction network of 50 SHC1-binding proteins is shown in Figure 11A. GEPIA2.0 tool was used to identify the top 100 genes that were correlated with SHC1 expression. SHC1 expression level was positively correlated with the expression of ADIPOR1 (*R* = 0.61), ARHGEF11 (*R* = 0.46), CHGB (*R* = 0.44), ITGB1 (*R* = 0.64) and NTRK1 (*R* = 0.38) genes (all *p* < 0.001) (Figure 11B).

The positive correlation between SHC1 and the above six genes in most cancers was expressed as a heatmap (Figure 11C). Intersection analysis was used to obtain two common members, ITGB1 and NTRK1 (Figure 11D). The two datasets from GEPIA and STRING were combined for KEGG and GO enrichment analyses. The KEGG data of Figure 6E suggested that SHC1 influences tumor pathogenesis *via* “PI3K-Akt signaling pathway,” “Proteoglycans in cancer,” “Focal adhesion,” “ErbB signaling pathway” and “EGFR tyrosine kinase inhibitor resistance.” The GO enrichment analysis further showed that most of these genes were linked to the biological process and cellular component. Besides, they were linked to molecular function of protein phosphorylation, such as peptidyl-tyrosine modification, peptidyl-tyrosine phosphorylation, protein autophosphorylation, protein tyrosine kinase activity, membrane raft, membrane microdomain, and membrane region (Figure 11F and Table 5).

**TABLE 5:** Results of GO-KEGG enrichment analysis.

Ontology	ID	Description	GeneRatio	BgRatio	*p*-value	*p*.Adjust	q-value
BP	GO:0018212	peptidyl-tyrosine modification	35/115	366/18670	3.22e−32	9.45e−29	6.04e−29
BP	GO:0018108	peptidyl-tyrosine phosphorylation	34/115	363/18670	5.77e−31	8.48e−28	5.42e−28
BP	GO:0014065	phosphatidylinositol 3-kinase signaling	21/115	148/18670	4.95e−23	4.85e−20	3.10e−20
BP	GO:0046777	protein autophosphorylation	24/115	235/18670	9.70e−23	7.13e−20	4.55e−20
BP	GO:0014068	positive regulation of phosphatidylinositol 3-kinase signaling	17/115	87/18670	2.68e−21	1.58e−18	1.01e−18
CC	GO:0045121	membrane raft	21/121	315/19717	3.87e−16	4.84e−14	3.39e−14
CC	GO:0098857	membrane microdomain	21/121	316/19717	4.12e−16	4.84e−14	3.39e−14
CC	GO:0098589	membrane region	21/121	328/19717	8.72e−16	6.83e−14	4.77e−14
CC	GO:0098802	plasma membrane receptor complex	13/121	295/19717	3.35e−08	1.97e−06	1.38e−06
CC	GO:0008305	integrin complex	5/121	31/19717	1.20e−06	4.48e−05	3.13e−05
MF	GO:0004713	protein tyrosine kinase activity	29/114	134/17697	7.63e−37	2.64e−34	2.10e−34
MF	GO:0004714	transmembrane receptor protein tyrosine kinase activity	19/114	62/17697	1.67e−27	2.88e−25	2.30e−25
MF	GO:0019199	transmembrane receptor protein kinase activity	20/114	79/17697	5.12e−27	5.90e−25	4.70e−25
MF	GO:0001784	phosphotyrosine residue binding	10/114	40/17697	5.93e−14	5.13e−12	4.09e−12
MF	GO:0043560	insulin receptor substrate binding	7/114	11/17697	1.23e−13	8.54e−12	6.81e−12
KEGG	hsa01521	EGFR tyrosine kinase inhibitor resistance	14/68	79/8076	1.79e−15	2.97e−13	1.21e−13
KEGG	hsa04012	ErbB signaling pathway	14/68	85/8076	5.26e−15	4.37e−13	1.77e−13
KEGG	hsa05205	Proteoglycans in cancer	18/68	205/8076	3.79e−14	1.82e−12	7.38e−13
KEGG	hsa04151	PI3K-Akt signaling pathway	22/68	354/8076	4.38e−14	1.82e−12	7.38e−13
KEGG	hsa04510	Focal adhesion	17/68	201/8076	4.04e−13	1.34e−11	5.44e−12

BP, biological process; CC, Cellular Component; MF, Molecular Function. *p* value <0.05 and q-value <0.25 were considered as significantly enriched.

## Discussion

Comprehensive methods such as pan-cancer data analysis can provide insights into clinical targets and treatment decisions and reveal similarities and differences in tumors. Moreover, they can identify key components driving metastasis, such as mutation spectrum, gene characteristics, related pathways, site-specificity, and disease gene phenotype (Ju et al., 2020a). In recent years, pan-cancer analysis based on multi-cancer species has attracted a lot of attention. Several pan-cancer studies have revealed that gene mutation, RNA alterations, immune infiltration, and driving genes are related to the occurrence and development of malignant tumors. Therefore, pan-cancer analysis is essential for the early diagnosis of tumors and the development of biomarkers. SHC1, a cytoplasmic signaling protein, plays a crucial role in transmitting receptor tyrosine kinase signals, insulin signaling, regulating reactive oxygen species (ROS), stress resistance, energy metabolism, cell apoptosis, and cell apoptosis senescence (Faisal et al., 2002; Bhat et al., 2015; Abou-Jaoude et al., 2018; Cao et al., 2018; Zhang et al., 2019; Hwang et al., 2020). Recent studies have shown that SHC1 is associated with several clinical diseases, especially in tumor development and progression (Falco et al., 2005; Smith et al., 2006; Ahn et al., 2017b; Chao et al., 2019b; Huang et al., 2019; Wright et al., 2019; Borah and Bhowmick, 2020; Kiepas et al., 2020). However, the roles of SHC1 in pan-cancer are unclear. Besides, it is unknown whether SHC1 plays a role in the pathogenesis of different tumors through some common molecular mechanisms.

This study comprehensively examined SHC1 expression levels and systematic prognostic landscape in genetic alteration, immune infiltration, DNA methylation, DNA mismatch repair, RNA methylation, or protein phosphorylation. SHC1 expression was significantly correlated with TMB/MSI, MMRs, DNA repair and methylation, and m6A RNA modification regulators in multiple cancers. SHC1 expression was also significantly associated with the clinical outcomes of CESC, KICH, KIRC, KIRP, LGG, LIHC, LUAD, and UVM patients. Moreover, altered SHC1 had a markedly worse prognosis of OS and DSS in SKCM. SHC1 expression was significantly correlated with immune cell infiltration levels, TAMs, and the expression of immune checkpoint markers, especially in HNSC, PRAD, and LGG. In addition, the results of the ROC analysis indicated the SHC1 exhibited strong diagnostic capability for KICH, LIHC, and PAAD. Altogether, these results suggest that SHC1 is a potential diagnosis and prognosis biomarker of certain tumors and can play a crucial role in tumor immunity.

The SHC1 gene encodes three isoform proteins p52Shc, p46Shc, and p66Shc. p52Shc and p46Shc are universally expressed, while p66Shc is expressed at different levels in different tissues (Bhat et al., 2015) (Pelicci et al., 1992b). A recent study indicated that SHC1 upregulation is correlated with poor OS, DFS, and early recurrence of HCC (Huang et al., 2019). Moreover, p66Shc is abnormally overexpressed in HCC cell lines (HuH7, HepG2, and SK-HEP1), and p46Shc and p52Shc are associated with HCC occurrence (Huang et al., 2019). p66Shc functions as a latent prognostic biomarker in breast cancer by inducing an epithelial-to-mesenchymal transition for more aggressive breast cancer (Hudson et al., 2014b). A previous study found that p52Shc promotes breast cancer initiation (Wright et al., 2019). Previous studies have shown that p66Shc tumor levels can act as a significant prognostic indicator of RFS and DSS in Stage IIA Colon Cancer (Grossman et al., 2007). Another study demonstrated that mir-5582-5p targets SHC1, inducing apoptosis and cell cycle arrest in colorectal cancer cells (An et al., 2016). Chao et al. (Chao et al., 2019b) recently found that RAB14 mediates SHC1 downregulation in human bladder cancer. A recent study also showed that upregulated SHC1 expression could act as a tumor promoter in bladder cancer and an underlying downstream of DEPDC1B (Lai et al., 2020b). Further, studies have shown that steroids upregulate p66Shc protein in hormone-sensitive cancer cells and primary prostate cancer tissue (Lee et al., 2004). p66Shc expression is also significantly upregulated in esophageal squamous cell carcinoma and adenocarcinoma (Bashir et al., 2010). Moreover, Zhao et al. (Zhao et al., 2020) indicated that SHC1 is significantly overexpressed in high-grade ccRCC and is correlated with poor prognosis.

This is the first study to show SHC1 is upregulated in brain and CNS cancers, head and neck cancers, kidney cancer, liver cancer, lung cancer, melanoma, and prostate melanoma using the Oncomine database. Moreover, the TIMER2.0 database showed that the mRNA level of SHC1 was significantly upregulated in BLCA, BRCA, CHOL, HNSC, KICH, LIHC, LUAD, LUSC, STAD, THCA, and KIRP. However, it was downregulated in KIRC, PRAD, and UCEC compared with the corresponding normal tissues. SHC1 expression was significantly higher in DLBC, GBM, PAAD, THYM, and PCPG, while lower in ACC and SKCM compared with the corresponding normal tissues (TCGA+GTEx datasets). Additionally, the expression of SHC1 total protein was significantly overexpressed in clear cell RCC and LUAD, while downregulated in UCEC (CPTAC dataset). Survival analysis of multiple databases showed that SHC1 expression was associated with poor prognosis in various cancers, especially in CESC, KICH, KIRC, KIRP, LGG, LIHC, LUAD, and UVM. These results are consistent with the previously observed carcinogenic effects of abnormal SHC1 expression. Together with the published literature, these data strongly suggest that SHC1 is a potential proto-oncogene and prognostic biomarker in CESC, KICH, KIRC, KIRP, LGG, LIHC, LUAD, and UVM.

Genetic alteration also affects the mRNA expression of genomic genes (Meng et al., 2020). Yang et al. recently found that the PLOD gene mutations alter its expression and are associated with a markedly poor prognosis in HCC patients (Yang et al., 2020). Another study also showed that CNV upregulation in KIRC *via* JAK3 is associated with high mRNA levels and short survival time (Liang et al., 2020). However, UCEC patients with SND1 alterations have better overall survival and disease-specific survival (Cui et al., 2020b). This study assessed the relationship between SHC1 gene mutation and the prognosis of Pan-cancer through the cBioPortal database. The genetic alteration of SHC1 was significantly correlated with the poor prognosis of OS and DSS in SKCM patients. This study assessed the potential association between SHC1 expression vs MSI, TMB, MMR gene mutation levels, and DNMT in all TCGA tumors. TMB and MSI are mainly associated with tumor immunotherapy response (Yarchoan et al., 2017; Zhao et al., 2019). SHC1 upregulation increased TMB in BRCA, KIRC, LGG, LUAD and THYM, and increased MSI in COAD, TGCT and UVM. MMR gene mutations predict tumorigenesis (Cerretelli et al., 2020). DNMTs alter chromatin structure and gene expression (Krzyzewska et al., 2019). Also, alterations in DNA methylation promote cancer development (Szigeti et al., 2018). SHC1 expression was associated with five MMR genes in human pan-cancers, especially in liver cancer. SHC1 expression was also positively correlated with three DNMTs (DNMT1, DNMT3A, and DNMT3B) in human cancers, especially in DLBC, KICH, KIRC, KIRP, LGG, LIHC, LUAD, MESO, TGCT, and UVM. RNA m6A is the methylation of N6 position of adenosine, which is the most common internal modification of mRNA and long noncoding RNA (lncRNA) in mammals. Previous studies have shown that abnormal methylation of m6A can promote or inhibit the expression of target genes, further affecting the development of breast cancer, lung cancer, liver cancer, colorectal cancer, leukemia and glioblastoma (Zhang, 2018). Herein, SHC1 expression was positively correlated with the expression of over 20 m6A RNA modification regulators in BRCA, COAD, DLBC, KIRP, LIHC, LUAD, OV, PRAD, PRAD, STAD, THCA, and UCEC. Taken together, these results show that abnormal expression of SHC1 can mediate tumorigenesis by regulating DNA damage, DNA methylation, or RNA methylation.

Tyrosine kinases and protein tyrosine phosphorylation also play a crucial role in tumor initiation and growth. SHCA is an adaptor protein that transmits extracellular signals downstream of receptor tyrosine kinases. Ursini-Siegel et al., (2008) reported that SHC1 contains PTB and SH2 phosphorylated tyrosine binding domains and three tyrosine-phosphorylated sites (Y239, Y240, and Y313). These are involved in the transduction of phosphorylated tyrosine dependent signals in breast cancer. A previous study showed that the SHCA tyrosine-phosphorylated sites are essential for PTB-independent SHCA pools to amplify breast tumor growth (Ha et al., 2018). Yukimasa et al. (Yoshida et al., 2013) also reported that p46Shc phosphorylation could indicate malignant transformation, tumor invasion, and metastasis in HCC. This study used the CPTAC dataset to explore the molecular mechanism of SHC1 protein expression in breast cancer, UCEC, ovarian cancer, lung adenocarcinoma, colon cancer, and clear cell RCC cancer based on total protein and phosphoprotein. The total SHC1 protein was significantly overexpressed in LUAD, and KIRC downregulated in UCEC than in normal controls. Moreover, the phosphorylation levels of SHC1protein were decreased in UCEC and colon cancer at S139, and breast, ovarian, and colon cancer at Y428. However, the phosphorylation levels of SHC1 were increased in clear cell RCC cancer at S139. Together with the existing researches, this study shows that SHC1 can act as an oncogene to regulate CCRCC tumorigenesis (Zhao et al., 2020). Therefore, SHC1 phosphorylation could be involved in the progression of clear cell RCC cancer. However, the relationship between reduced phosphorylation levels of SHC1, as a potential oncogenic factor, and the progression and prognosis of other cancers is unknown. Therefore, further experiments are needed to evaluate the potential role of SHC1 phosphorylation and related regulatory mechanisms in tumorigenesis.

Tumor microenvironment, especially the tumor immune microenvironment, plays a vital role in tumor occurrence and progression. Several studies have revealed that a comprehensive evaluation of tumor-infiltrating immune cells (TICs) can provide a clinical outcome for immunotherapy (Bai et al., 2020; Ju et al., 2020b; Guo et al., 2020). This study also assessed the correlation between the SHC1 expression and immune cell infiltration levels. SHC1 expression was positively correlated with the infiltration levels of six immune cells in various cancers, especially in PRAD. Also, several immune infiltrating cells can be independent predictors of prognosis in cancer patients. Another study also suggested that immune cells are essential stromal compartments in the TME that affect patients’ survival (Bonanno et al., 2018). Kaplan-Meier analysis was used to determine the correlation between the aberrant SHC1 expression and immune cell infiltration with clinical performance. Herein, high or low levels of SHCI expression and high or low immune cell infiltrations had different tumor outcomes, especially in BLCA, BRCA, COAD, LIHC, LUAD, LGG, PAAD, SKCM, SARC, and STAD. A previous study showed that tyrosine kinases require downstream SHC1 signaling to evade anti-tumor immunity in breast cancer (Ursini-Siegel et al., 2010). Another study also found that the SHCA phosphotyrosine motif enhances immune suppression by inhibiting multiple signals and participates in the immune escape of tumors (Ahn et al., 2017b). Macrophages are widely considered to be the first line of defense against tumor immunity. Herein, BRCA patients with high expression of SHC1 and macrophage infiltration had poor overall survival (OS). Taken together, these results show that SHC1 plays a key role in immune escape and immunosuppression in breast cancer. Immune checkpoints inhibit the over-activation of the active immune system. Tumor cells evade cellular immunity by activating immune checkpoints to suppress immune responses. Moreover, immune checkpoint molecules are overexpressed or enhanced in various diseases, such as tumors. Herein, SHC1 was more or less co-expressed with various immune checkpoint molecules in tumors, especially in HNSC, LUAD, SARC and TGCT. SHC1 was positively correlated with infiltration levels of macrophages in most tumors. Several evidence have demonstrated that infiltration of TAMs promotes tumor progression and is closely associated with the immunosuppressive state of the tumor (Shiau et al., 2020; Wu et al., 2020; Ngambenjawong et al., 2017). M0 and M2 infiltration levels were positively correlated with SHC1 expression in most tumors, especially in COAD, HNSC, LGG, LUSC, and READ. These findings suggest that high SHC1 expression is associated with tumor immune cell infiltration and affects the prognosis of patients. High SHC1 expression is closely associated with the immunosuppressive state of tumors, providing a potential target for immunotherapy.

A comprehensive analysis of the diagnostic value of SHC1 in pan-cancer was also conducted. KEGG and GO enrichment analyses showed the potential impact of the “PI3K-Akt signaling pathway,” “ErbB signaling pathway,” “Focal adhesion,” and “Proteoglycans in cancer” or “EGFR tyrosine kinase inhibitor resistance” of cancers.

Our study has several limitations. Firstly, the mRNA and protein levels of SHC1 are assessed in our study, while its levels need to be further validated by human tissue in future studies. Secondly, additional validation in prognostic and diagnostic values from public datasets is required to support our present results. Thirdly, as multiple information from diverse databases was retrieved for the analysis, systematic bias exists. More efforts are needed to explore the role of SHC1 in cancer and the value of SHC1 as a potential target of anticancer therapy.

Taken together, our first pan-cancer study indicated that SHC1 expression was correlated with clinical outcomes, genetic mutation, TMB, MSI, DNA methylation, RNA methylation, protein phosphorylation, immune cell infiltration, and tumor-associated macrophage. This study also outlines the diagnostic efficacy of SCH1 in multiple tumors. All these findings will help understand the role of SHC1 in tumorigenesis.

## Conclusion

In summary, these findings highlight that SHC1 plays an important role in the tumor immune microenvironment, and SHC1 has been identified to have prognostic and diagnostic value in multiple cancers. Thus, SHC1 as an effective prognostic and diagnostic biomarker may play a key role as a potential target for cancer immunotherapy.

## Data Availability

The original contributions presented in the study are included in the article/Supplementary Material, further inquiries can be directed to the corresponding authors.

## Glossary

ACC: Adrenocortical carcinoma
ADIPOR1: Adiponectin receptor 1
ARHGEF11: Rho guanine nucleotide exchange factor (GEF) 11
AUC: Area under the curve
BLCA: bladder urothelial carcinoma
BRCA: breast invasive carcinoma
CHGB: chromogranin B
CHOL: cholangiocarcinoma
COAD: colon adenocarcinoma
DFS: disease-free survival
DLBC: Lymphoid Neoplasm Diffuse Large B-cell Lymphoma
DMFS: distant metastasis-free survival
DNMT: DNA methyltransferases
DSS: disease-specific survival
ESCA: Esophageal carcinoma
GBM: Glioblastoma multiforme
GEO: Gene Expression Omnibus
GTEx: Genotype Tissue Expression dataset
HNSC: head and neck squamous cell carcinoma
HNSC-HPVpos: head and neck squamous carcinoma- HPV positive
HR: hazard ratio
ITGB1: Integrin, beta 1
KICH: kidney chromophobe
KIRC: kidney renal clear cell carcinoma
LAML: Acute Myeloid Leukemia
LGG: brain lower grade glioma
LIHC: liver hepatocellular carcinoma
LUAD: lung adenocarcinoma
LUSC: lung squamous cell carcinoma
MMR: DNA Mismatch repair
MSI: microsatellite instability
NTRK: Neurotrophic tyrosine kinase, receptor, type 1
OS: overall survival
OV: ovarian cancer
PAAD: Pancreatic adenocarcinoma
PCPG: Pheochromocytoma and Paraganglioma
PFS: progression-free survival
PRAD: prostate adenocarcinoma
READ: rectum adenocarcinoma
RFS: relapse-free survival
ROC: Receiver-operating characteristic
SARC: Sarcoma
SKCM: Skin Cutaneous Melanoma
ssGSEA: Single sample gene set enrichment analysis
STAD: Stomach adenocarcinoma
TAM: tumor-associated-macrophage
TCGA: The Cancer Genome Atlas
TGCT: Testicular Germ Cell Tumor
THCA: Thyroid carcinoma
THYM: Thymoma
TMB: Tumor mutation burden
TME: tumor microenvironment
UCEC: uterine corpus endometrial carcinoma
UCS: Uterine Carcinosarcoma
UVM: Uveal Melanoma
Oncomine database: https://www.oncomine.org/resource/login.html
TCGA Data Portal: https://portal.gdc.cancer.gov/
TIMER2: http://timer.cistrome.org/
GEPIA2: http://gepia2.cancer-pku.cn/#analysis
PrognoScan: http://dna00.bio.kyutech.ac.jp/PrognoScan/index.html
UALCAN portal: http://ualcan.path.uab.edu/analysis-prot.html
cBioPortal: https://www.cbioportal.org/
STRING website: https://string-db.org/
GEO Datasets: https://www.ncbi.nlm.nih.gov/gds/
KM-plotter: http://kmplot.com/analysis/

## References

[B1] Davis A. A. Chae Y. K. Agte S. Pan A. Giles F. J. (2017), Association of Tumor Mutational burden (TMB) with DNA Repair Mutations and Response to Anti-PD-1/pd-L1 Therapy in Non-small Cell Lung Cancer (NSCLC). 20(2):88–96. 10.1016/j.cllc.2018.09.008 30425022

[B2] Abou-Jaoude A. Badiqué L. Mlih M. Awan S. Guo S. Lemle A. (2018). Author Correction: Loss of the Adaptor Protein ShcA in Endothelial Cells Protects against Monocyte Macrophage Adhesion, LDL-Oxydation, and Atherosclerotic Lesion Formation. Sci. Rep. 8, 9577. 10.1038/s41598-018-27564-1 29921842 PMC6008455

[B3] Ahn R. Sabourin V. Bolt A. M. Hébert S. Totten S. De Jay N. (2017). The Shc1 Adaptor Simultaneously Balances Stat1 and Stat3 Activity to Promote Breast Cancer Immune Suppression. Nat. Commun. 8, 14638. 10.1038/ncomms14638 28276425 PMC5347092

[B4] Ahn R. Sabourin V. Bolt A. M. Hébert S. Totten S. De Jay N. (2017). The Shc1 Adaptor Simultaneously Balances Stat1 and Stat3 Activity to Promote Breast Cancer Immune Suppression. Nat. Commun. 8, 14638. 10.1038/ncomms14638 28276425 PMC5347092

[B5] An H.-J. Kwak S.-Y. Yoo J.-O. Kim J.-S. Bae I.-H. Park M.-J. (2016). Novel miR-5582-5p Functions as a Tumor Suppressor by Inducing Apoptosis and Cell Cycle Arrest in Cancer Cells through Direct Targeting of GAB1, SHC1, and CDK2. Biochim. Biophys. Acta (Bba) - Mol. Basis Dis. 1862, 1926–1937. 10.1016/j.bbadis.2016.07.017 27475256

[B6] Aran D. Butte A. J. (2016). Digitally Deconvolving the Tumor Microenvironment. Genome Biol. 17, 175. 10.1186/s13059-016-1036-7 27549319 PMC4993002

[B7] Bai X. Wu D. H. Ma S. C. Tang X-R Kangn S. Wang J. (2020), Development and validation of a genomic mutation signature to predict response to PD-1 inhibitors in non-squamous NSCLC: a multicohort study, J Immunother Cancer 8, e000381. 10.1136/jitc-2019-000381 32606052 PMC7328897

[B8] Bardou P. Mariette J. Escudié F. Djemiel C. Klopp C. J. B. B. (2014). Jvenn: an Interactive Venn Diagram Viewe. BMC Bioinformatics 15, 293. 10.1186/1471-2105-15-293 25176396 PMC4261873

[B9] Bashir M. Kirmani D. Bhat H. F. Baba R. A. Hamza R. Naqash S. (2010). P66shc and its Downstream Eps8 and Rac1 Proteins Are Upregulated in Esophageal Cancers. Cell Commun Signal 8, 13. 10.1186/1478-811x-8-13 20565814 PMC2901305

[B10] Bhat S. S. Anand D. Khanday F. A. (2015). p66Shc as a Switch in Bringing about Contrasting Responses in Cell Growth: Implications on Cell Proliferation and Apoptosis. Mol. Cancer 14, 76. 10.1186/s12943-015-0354-9 25890053 PMC4421994

[B11] Blum A. Wang P. Zenklusen J. C. (2018). SnapShot: TCGA-Analyzed Tumors. Cell 173, 530. 10.1016/j.cell.2018.03.059 29625059

[B12] Bonanno L. Pavan A. Dieci M. V. Di Liso E. Schiavon M. Comacchio G. (2018). The Role of Immune Microenvironment in Small-Cell Lung Cancer: Distribution of PD-L1 Expression and Prognostic Role of FOXP3-Positive Tumour Infiltrating Lymphocytes. Eur. J. Cancer 101, 191–200. 10.1016/j.ejca.2018.06.023 30077124

[B13] Borah S. Bhowmick N. A. (2020). The Adaptor Protein SHCA Launches Cancer Invasion. J. Biol. Chem. 295, 10560–10561. 10.1074/jbc.h120.014283 32737145 PMC7397101

[B14] Campbell K. S. Ogris E. Burke B. Su W. Auger K. R. Druker B. J. (1994). Polyoma Middle Tumor Antigen Interacts with SHC Protein via the NPTY (Asn-Pro-Thr-Tyr) Motif in Middle Tumor Antigen. Proc. Natl. Acad. Sci. 91, 6344–6348. 10.1073/pnas.91.14.6344 8022784 PMC44198

[B15] Cao W. Liu X. Xu X. Zeng M. Sun B. Yu X. (2018). The Src Homology and Collagen A (ShcA) Adaptor Protein May Participate in the Pathogenesis of Membranous Lupus Nephritis. Lupus 27, 2014–2019. 10.1177/0961203318796295 30189773

[B16] Carrato C. Alameda F. Esteve-Codina A. Pineda E. Arpí O. Martinez-García M. (2020). Glioblastoma TCGA Mesenchymal and IGS 23 Tumors Are Identifiable by IHC and Have an Immune-Phenotype Indicating a Potential Benefit from Immunotherapy. Clin. Cancer Res. 26, 6600–6609. 10.1158/1078-0432.ccr-20-2171 32998960

[B17] Cerretelli G. Ager A. Frayling I. A. M Arends M. J. (2020). Molecular pathology of Lynch syndromeI. J Pathol. 250, 518-531. 10.1002/path.5422 32141610

[B18] Chandrashekar D. S. Bashel B. Balasubramanya S. A. H. Creighton C. J. Ponce-Rodriguez I. Chakravarthi B. V. S. K. (2017). UALCAN: A Portal for Facilitating Tumor Subgroup Gene Expression and Survival Analyses. Neoplasia 19, 649–658. 10.1016/j.neo.2017.05.002 28732212 PMC5516091

[B19] Chao H. Deng L. Xu F. Fu B. Zhu Z. Dong Z. (2019). RAB14 Activates MAPK Signaling to Promote Bladder Tumorigenesis. Carcinogenesis 40, 1341–1351. 10.1093/carcin/bgz039 30809635

[B20] Cho M-Y. Park M-J. Kwak S-Y. Kim J. An H.-.J. Kim J-S. (2016). Novel miR-5582-5p Functions as a Tumor Suppressor by Inducing Apoptosis and Cell Cycle Arrest in Cancer Cells through Direct Targeting of GAB1, SHC1, and CDK2. Biochim. Biophys. Acta (Bba) - Mol. Basis Dis. 1862 (10), 1926–1937. 10.1016/j.bbadis.2016.07.017 27475256

[B21] Choi K. Y. Cho Y. J. Kim J. S. Ahn Y.-H. Hong S. H. Communications B. R. (2015). SHC1 Sensitizes Cancer Cells to the 8-Cl-cAMP Treatment. Biochem. Biophysical Res. Commun. 463, 673–678. 10.1016/j.bbrc.2015.05.123 26043699

[B22] Cui X. Zhang X. Liu M. Zhao C. Yang J. J. G. (2020). A pan-cancer Anal. oncogenic role staphylococcal nuclease domain-containing Protein 1 (Snd1) Hum. tumors, Genomics 112:3958-3967. 10.1016/j.ygeno.2020.06.044 32645525

[B23] Cui X. Zhang X. Liu M. Zhao C. Zhang N. Ren Y. (2020). A Pan-Cancer Analysis of the Oncogenic Role of Staphylococcal Nuclease Domain-Containing Protein 1 (SND1) in Human Tumors. Genomics 112, 3958–3967. 10.1016/j.ygeno.2020.06.044 32645525

[B24] Shiau D. J. Kuo W. T. Davuluri G. Shieh C. C. Tsai P. J. Chen C. C. (2020), Hepatocellular Carcinoma-Derived High Mobility Group Box 1 Triggers M2 Macrophage Polarization via a TLR2/NOX2/autophagy axis. Sci Rep 10(1):13582. 10.1038/s41598-020-70137-4 32788720 PMC7423894

[B25] Clough E. Barrett T.J.o (2016), the Gene Expression Omnibus Database. Methods Mol Biol. 1418:93-110. 10.1007/978-1-4939-3578-9_5 27008011 PMC4944384

[B26] Faisal A. M. El-Shemerly M. Hess D. Nagamine Y. (2002). Serine/Threonine Phosphorylation of ShcA. J. Biol. Chem. 277, 30144–30152. 10.1074/jbc.m203229200 12052829

[B27] Falco V. D. Guarino V. Malorn L. Cirafici M. C. Troglio F. Erreni M. (2005), RAI(ShcC/N-Shc)-dependent Recruitment of GAB 1 to RET Oncoproteins Potentiates PI 3-K Signalling in Thyroid Tumors. J. Oncogene. 24(41):6303–6313. 10.1038/sj.onc.1208776 15940252

[B28] Gao J. Aksoy B. Dogrusoz U. Dresdner G. Gross B. Sumer S. (2013). N.J.S.s. Schultz. Integrative analysis of complex cancer genomics and clinical profiles using the cBioPortal, Sci Signal 6, pl1. 10.1126/scisignal.2004088 PMC4160307 23550210

[B29] Grossman S. R. Lyle S. Resnick M. B. Sabo E. Lis R. T. Rosinha E. (2007). p66 Shc Tumor Levels Show a Strong Prognostic Correlation with Disease Outcome in Stage IIA Colon Cancer. Clin. Cancer Res. 13, 5798–5804. 10.1158/1078-0432.ccr-07-0073 17908971

[B30] Guo L. Li X. Liu R. Chen Y. Ren C. Du S. (2020). TOX Correlates with Prognosis, Immune Infiltration, and T Cells Exhaustion in Lung Adenocarcinoma. Cancer Med. 9, 6694–6709. 10.1002/cam4.3324 32700817 PMC7520261

[B31] Ha J. R. Ahn R. Smith H. W. Sabourin V. Hébert S. Cepeda Cañedo E. (2018). Integration of Distinct ShcA Signaling Complexes Promotes Breast Tumor Growth and Tyrosine Kinase Inhibitor Resistance. Mol. Cancer Res. 16, 894–908. 10.1158/1541-7786.mcr-17-0623 29453318

[B32] Chao H. Deng L. Xu F. Fu B. Zhu Z. Dong Z. (2019), RAB14 Activates MAPK Signaling to Promote Bladder Tumorigenesis. 11. 10.1093/carcin/bgz039 30809635

[B33] He Q. Qin S. Tao L. Ning H. Jiang H. J. O. l. (2019). Prognostic Value and Prospective Molecular Mechanism of miR-100-5p in Hepatocellular Carcinoma: A Comprehensive Study Based on 1,258 Samples. Oncol. Lett. 18, 6126–6142. 31788087 10.3892/ol.2019.10962 PMC6865135

[B34] Hellmann M. Ciuleanu T. E. Pluzanski A. Lee J. S. Otterson G. A. Audigier-Valette C. (2018). Nivolumab Plus Ipilimumab in Lung Cancer with a High Tumor Mutational Burden. N. Engl. J. Med. 378 (22), 2093–2104. 10.1056/NEJMoa1801946 29658845 PMC7193684

[B35] Hu B. Yang X.-B. Sang X.-T. (2020). Development of an Immune-Related Prognostic index Associated with Hepatocellular Carcinoma. Aging 12, 5010–5030. 10.18632/aging.102926 32191631 PMC7138589

[B36] Huang P. Feng X. Zhao Z. Yang B. Fang T. Guo M. (2019). p66Shc Promotes HCC Progression in the Tumor Microenvironment via STAT3 Signaling. Exp. Cel Res. 383, 111550. 10.1016/j.yexcr.2019.111550 31398350

[B37] Hudson J. Ha J. R. Sabourin V. Ahn R. La Selva R. Livingstone J. (2014). p66ShcA Promotes Breast Cancer Plasticity by Inducing an Epithelial-To-Mesenchymal Transition. Mol. Cell Biol. 34, 3689–3701. 10.1128/mcb.00341-14 25071152 PMC4187732

[B38] Hudson J. Ha J. R. Sabourin V. Ahn R. Selva R. L. Livingstone J. (2014). p66ShcA Promotes Breast Cancer Plasticity by Inducing an Epithelial-To-Mesenchymal Transition. Mol. Cell Biol. 34 (19), 3689–3701. 10.1128/mcb.00341-14 25071152 PMC4187732

[B39] Hwang J. Shin N. Shin H. Yin Y. Kwon H. Park H. (2020), Protective Effects of ShcA Protein Silencing for Photothrombotic Cerebral Infarction. 12(5):866–878. 10.1007/s12975-020-00874-1 33242144

[B40] Ju Q. Li X. Zhang H. Yan S. Zhao Y Li Y. (2020). NFE2L2 Is a Potential Prognostic Biomarker and Is Correlated with Immune Infiltration in Brain Lower Grade Glioma: A Pan-Cancer Analysis, Oxid Med Cell Longev 2020, 3580719. 10.1155/2020/3580719 33101586 PMC7569466

[B41] Ju Q. Li X. Zhang H. Yan S. Li Y. Zhao Y. (2020). NFE2L2 Is a Potential Prognostic Biomarker and Is Correlated with Immune Infiltration in Brain Lower Grade Glioma: A Pan-Cancer Analysis. Oxidative Med. Cell LongevityOxid Med Cel Longev 2020, 1–26. 10.1155/2020/3580719 PMC7569466 33101586

[B42] Kiepas A. Voorand E. Senecal J. Ahn R. Annis M. G. Jacquet K. (2020). The SHCA Adapter Protein Cooperates with Lipoma-Preferred Partner in the Regulation of Adhesion Dynamics and Invadopodia Formation. J. Biol. Chem. 295, 10535–10559. 10.1074/jbc.ra119.011903 32299913 PMC7397114

[B43] Krzyzewska I. M. Maas S. M. Henneman P. Lip K. Mannens M. J. C. E. (2019). A genome-wide DNA methylation signature for SETD1B-related syndrome, Clin Epigenetics 11, 156. 10.1186/s13148-019-0749-3 31685013 PMC6830011

[B44] Lai C.-H. Xu K. Zhou J. Wang M. Zhang W. Liu X. (2020). DEPDC1B Is a Tumor Promotor in Development of Bladder Cancer through Targeting SHC1. Cell Death Dis 11, 986. 10.1038/s41419-020-03190-6 33203836 PMC7672062

[B45] Lai C. H. Xu K. Zhou J. Wang M. Hu H. J. C. D. (2020). Disease. DEPDC1B is a tumor promotor in development of bladder cancer through targeting SHC1, Cell Death Dis 11:986. 10.1038/s41419-020-03190-6 PMC7672062 33203836

[B46] Lánczky A. Nagy Á. Bottai G. Munkácsy G. Szabó A. Santarpia L. (2016). miRpower: a Web-Tool to Validate Survival-Associated miRNAs Utilizing Expression Data from 2178 Breast Cancer Patients. Breast Cancer Res. Treat. 160, 439–446. 10.1007/s10549-016-4013-7 27744485

[B47] Lee M.-S. Igawa T. Chen S.-J. Van Bemmel D. Lin J. S. Lin F.-F. (2004). p66Shc Protein Is Upregulated by Steroid Hormones in Hormone-Sensitive Cancer Cells and in Primary Prostate Carcinomas. Int. J. Cancer 108, 672–678. 10.1002/ijc.11621 14696093

[B48] Lewis K. Kiepas A. Hudson J. Senecal J. Ha J. R. Voorand E. (2020). p66ShcA Functions as a Contextual Promoter of Breast Cancer Metastasis. Breast Cancer Res. 22, 7. 10.1186/s13058-020-1245-6 31941526 PMC6964019

[B49] Liang F. Liang H. Li Z. Huang P. (2020). JAK3 Is a Potential Biomarker and Associated with Immune Infiltration in Kidney Renal clear Cell Carcinoma. Int. Immunopharmacology 86, 106706. 10.1016/j.intimp.2020.106706 32570038

[B50] Liu J. Zhang S. Dai W. Xie C. Li J-C. (2020). A Comprehensive Prognostic and Immune Analysis of SLC41A3 in Pan-Cancer. Front. Oncol. 10, 586414. 10.3389/fonc.2020.582667 33520701 PMC7841432

[B51] Wu J Gao W Tang Q Yu Y You W Wu Z (2020), M2 Macrophage-Derived Exosomes Facilitate Hepatocarcinoma Metastasis by Transferring αMβ2 Integrin to Tumor Cells, J. Hepatol. 74(6):3564. 10.1002/hep.32229 PMC9050086 34850979

[B52] Meng J. Lu X. Zhou Y. Zhang M. Gao L. Gao S. (2020). Characterization of the Prognostic Values and Response to Immunotherapy/chemotherapy of Krüppel‐like Factors in Prostate Cancer. J. Cel Mol Med 24, 5797–5810. 10.1111/jcmm.15242 PMC7214179 32281273

[B53] Miller D. R. Ingersoll M. A. Chatterjee A. Baker B. Shrishrimal S. Kosmacek E. A. (2019). p66Shc Protein through a Redox Mechanism Enhances the Progression of Prostate Cancer Cells towards Castration-Resistance. Free Radic. Biol. Med. 139, 24–34. 10.1016/j.freeradbiomed.2019.05.015 31100478 PMC6620027

[B54] Mir H. A. Ali R. Mushtaq U. Khanday F. A. (2020). Structure-functional Implications of Longevity Protein p66Shc in Health and Disease. Ageing Res. Rev. 10.1016/j.arr.2020.101139 32795504

[B55] Mizuno H. Kitada K. Nakai K. Sarai A. (2009). PrognoScan: a New Database for Meta-Analysis of the Prognostic Value of Genes. BMC Med. Genomics 2, 18. 10.1186/1755-8794-2-18 19393097 PMC2689870

[B56] Morais-Rodrigues F. Silv́erio-Machado R. Kato R. B. Rodrigues D. L. N. Valdez-Baez J. Fonseca V. (2020). Analysis of the Microarray Gene Expression for Breast Cancer Progression after the Application Modified Logistic Regression. Gene 726, 144168. 10.1016/j.gene.2019.144168 31759986

[B57] Ngambenjawong C. Gustafson H. H. Pun S. H. J. A. D. D. R. (2017). Progress in Tumor-Associated Macrophage ( TAM )-targeted Therapeutics. Adv. Drug Deliv. Rev. 114, 206–221. 10.1016/j.addr.2017.04.010 28449873 PMC5581987

[B58] Pelicci G. Lanfrancone L. Grignani F. Mcglade J. Cavallo F. Forni G. (1992). A Novel Transforming Protein (SHC) with an SH2 Domain Is Implicated in Mitogenic Signal Transduction. Cell 70, 93–104. 10.1016/0092-8674(92)90536-l 1623525

[B59] Pelicci G. Lanfrancone L. Grignani F. McGlade J. Cavallo F. Forni G. (1992). A Novel Transforming Protein (SHC) with an SH2 Domain Is Implicated in Mitogenic Signal Transduction. Cell 70, 93–104. 10.1016/0092-8674(92)90536-l 1623525

[B60] Rhodes D. R. Kalyana-Sundaram S. Mahavisno V. Varambally R. Yu J. Briggs B. B. (2007). Oncomine 3.0: Genes, Pathways, and Networks in a Collection of 18,000 Cancer Gene Expression Profiles. Neoplasia 9, 166–180. 10.1593/neo.07112 17356713 PMC1813932

[B61] Robin X. Turck N. Hainard A. Tiberti N. Lisacek F. Sanchez J.-C. (2011). pROC: an Open-Source Package for R and S+ to Analyze and Compare ROC Curves. BMC Bioinformatics 12, 77. 10.1186/1471-2105-12-77 21414208 PMC3068975

[B62] Smith S. M. Crowe D. L. Lee M. K. (2006). β1 Integrins Modulate p66ShcA Expression and EGF-Induced MAP Kinase Activation in Fetal Lung Cells. Biochem. Biophysical Res. Commun. 342, 909–918. 10.1016/j.bbrc.2006.02.058 16517240

[B63] Szigeti K. A. Galamb O. Kalmár A. Barták B. K. Nagy Z. B. Márkus E. (2018). Role and Alterations of DNA Methylation during the Aging and Cancer. Orv Hetil 159, 3–15. 10.1556/650.2018.30927 29291647

[B64] Tang Z. Kang B. Li C. Chen T. Zhang Z. (2019). GEPIA2: an Enhanced Web Server for Large-Scale Expression Profiling and Interactive Analysis. Nucleic Acids Res. 47, W556–W560. 10.1093/nar/gkz430 31114875 PMC6602440

[B65] Ursini-Siegel J. Cory S. Zuo D. Hardy W. R. Rexhepaj E. Lam S. (2010). Receptor Tyrosine Kinase Signaling Favors a Protumorigenic State in Breast Cancer Cells by Inhibiting the Adaptive Immune Response. Cancer Res. 70, 7776–7787. 10.1158/0008-5472.can-10-2229 20924104 PMC3660232

[B66] Ursini-Siegel J. Muller W. J. (2008). The ShcA Adaptor Protein Is a Critical Regulator of Breast Cancer Progression. Cell Cycle 7, 1936–1943. 10.4161/cc.7.13.6205 18604176

[B67] Wickham H. (2016), ggplot2, Elegant Graphics for Data Analysis, Springer Nature. 10.1007/978-3-319-24277-4

[B68] Wright K. D. Miller B. S. El-Meanawy S. Tsaih S.-W. Banerjee A. Geurts A. M. (2019). The P52 Isoform of SHC1 Is a Key Driver of Breast Cancer Initiation. Breast Cancer Res. 21, 74. 10.1186/s13058-019-1155-7 31202267 PMC6570928

[B69] Yang B. Zhao Y. Wang L. Zhao Y. Wei L. Chen D. (2020). Identification of PLOD Family Genes as Novel Prognostic Biomarkers for Hepatocellular Carcinoma. Front. Oncol. 10, 1695. 10.3389/fonc.2020.01695 33014843 PMC7509443

[B70] Yang Y. Wu G. Li Q. Zheng Y. Liu M. Zhou L. (2021). Angiogenesis-Related Immune Signatures Correlate with Prognosis, Tumor Microenvironment, and Therapeutic Sensitivity in Hepatocellular Carcinoma. Front. Mol. Biosci. 8, 690206. 10.3389/fmolb.2021.690206 34262941 PMC8273615

[B71] Yarchoan M. Hopkins A. Jaffee E. M. (2017). Tumor Mutational Burden and Response Rate to PD-1 Inhibition. N. Engl. J. Med. 377, 2500–2501. 10.1056/nejmc1713444 29262275 PMC6549688

[B72] Liang Y. Lei Y. Du M. Liang M. Gao Y. J. J. o. C. Medicine M., The Increased Expression and Aberrant Methylation of SHC1 in Non–small Cell Lung Cancer: Integrative Analysis of Clinical and Bioinformatics Databases. (2021). 10.1111/jcmm.16717 PMC8278126 34117717

[B73] Yoshida S. Kornek M. Ikenaga N. Schmelzle M. Masuzaki R. Csizmadia E. (2013). Sublethal Heat Treatment Promotes Epithelial-Mesenchymal Transition and Enhances the Malignant Potential of Hepatocellular Carcinoma. Hepatology 58, 1667–1680. 10.1002/hep.26526 23729316

[B74] Yu G. Wang L.-G. Han Y. He Q.-Y. (2012). clusterProfiler: an R Package for Comparing Biological Themes Among Gene Clusters. OMICS: A J. Integr. Biol. 16, 284–287. 10.1089/omi.2011.0118 PMC3339379 22455463

[B75] Zhang S. J. (2018), Mechanism of N 6 -methyladenosine Modification and its Emerging Role in Cancer. Pharmacol Ther. 189:173-183. 10.1016/j.pharmthera.2018.04.011 29730276

[B76] Zhang T. Zhao X. Hai R. Li R. Zhang W. Zhang J. (2019). p66Shc Is Associated with Hydrogen Peroxide-Induced Oxidative Stress in Preimplantation Sheep Embryos. Mol. Reprod. Dev. 86, 342–350. 10.1002/mrd.23110 30636355

[B77] Zhao P. Li L. Jiang X. Li Q. (2019). Mismatch Repair Deficiency/microsatellite Instability-High as a Predictor for Anti-PD-1/pd-L1 Immunotherapy Efficacy. J. Hematol. Oncol. 12, 54. 10.1186/s13045-019-0738-1 31151482 PMC6544911

[B78] Zhao Y. Wang Y. Zhao E. Tan Y. Geng B. Kang C. (2020). PTRF/CAVIN1, Regulated by SHC1 through the EGFR Pathway, Is Found in Urine Exosomes as a Potential Biomarker of ccRCC. Carcinogenesis 41, 274–283. 10.1093/carcin/bgz147 31605605

