# Supplementary figures and images for: Pan-Cancer Study of SHC-Adaptor Protein 1 (SHC1) as a Diagnostic, Prognostic and Immunological Biomarker in Human Cancer
Source: Front Genet. 2022 May 2;13:817118. doi: 10.3389/fgene.2022.817118 (PMC9115805; doi:10.3389/fgene.2022.817118)

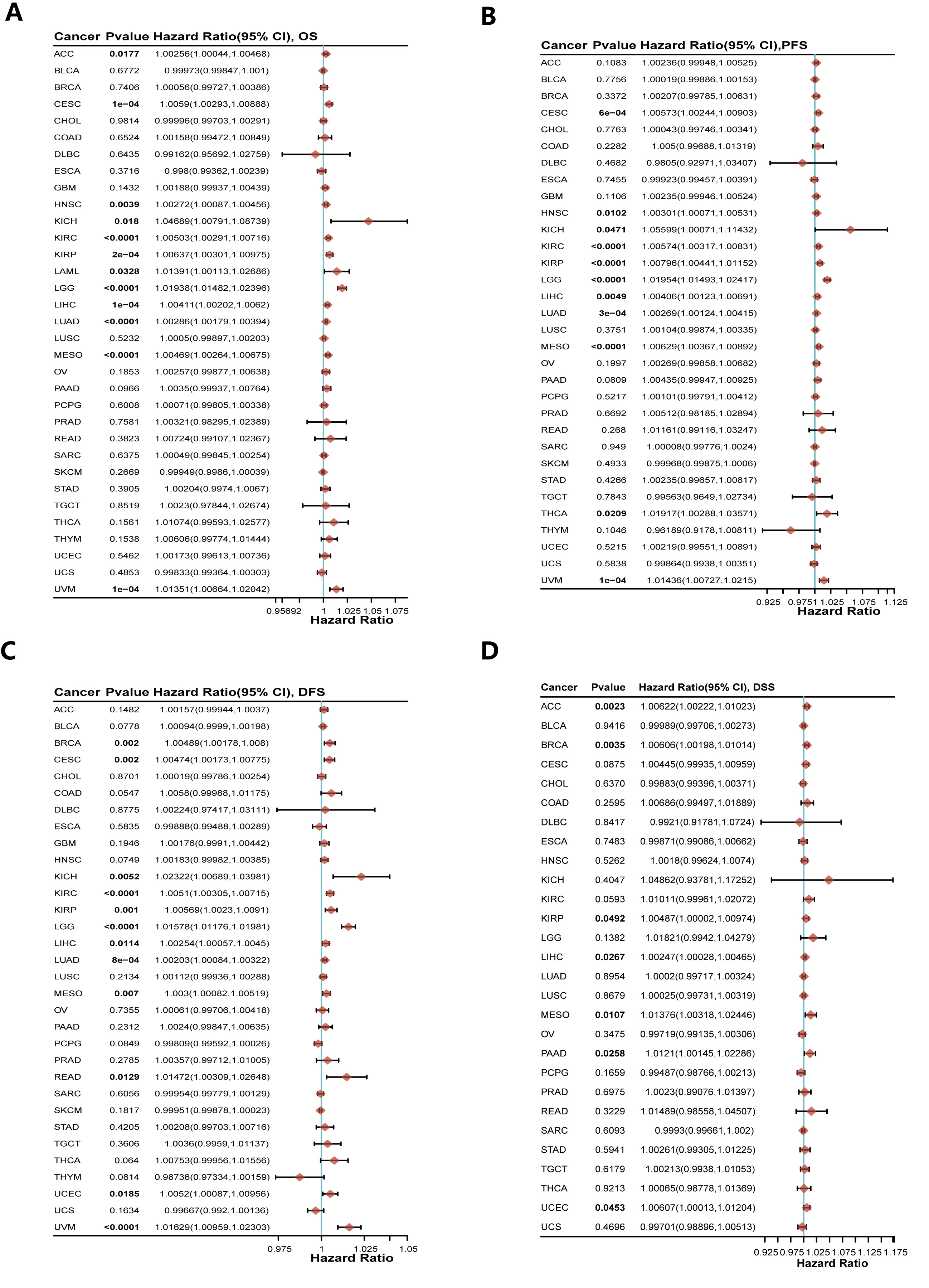

Supplement: Supplementary file 1 [file Image1.JPEG]
